# The Hippo effector TEAD1 regulates postnatal murine cerebellar development

**DOI:** 10.1007/s00429-025-02903-x

**Published:** 2025-03-10

**Authors:** Cooper Atterton, Alexandra Pelenyi, Justin Jones, Laura Currey, Majd Al-Khalily, Lucinda Wright, Mikki Doonan, David Knight, Nyoman D. Kurniawan, Shaun Walters, Stefan Thor, Michael Piper

**Affiliations:** 1 The School of Biomedical Sciences, Faculty of Medicine, The University of Queensland, Brisbane, QLD 4072 Australia; 2 The Centre for Advanced Imaging, The University of Queensland, Brisbane, QLD 4072 Australia; 3 The Queensland Brain Institute, The University of Queensland, Brisbane, QLD 4072 Australia

**Keywords:** TEAD1, Hippo, Cerebellum, Granule cell, Purkinje neuron, Bergmann glia

## Abstract

The Hippo signalling cascade is an evolutionarily conserved pathway critical for the development of numerous organ systems and is required for the development of many parts of the mammalian nervous system, including the cerebellum. The Hippo pathway converges, via the nuclear YAP/TAZ co-transcription factors, on transcription factors of the TEA Domain (TEAD) family (TEAD1-4) and promotes the expression of pro-proliferative genes. Despite the importance of TEAD function, our understanding of spatial and temporal expression of this family is limited, as is our understanding of which TEAD family members regulate Hippo-dependent organ development. Here, we focus on TEAD1 and how this factor contributes to postnatal murine cerebellar development. We find expression of TEAD1 within cerebellar progenitor cells and glial cells, including astrocytes and Bergmann glia, as well as by some interneurons within the granular layer. The importance of TEAD1 expression for cerebellar development was investigated using a conditional ablation approach, which revealed a range of developmental deficits in *Tead1* mutants, including an underdeveloped cerebellum, morphological defects in Bergmann Glia and Purkinje Neurons, as well as granule neuron migration defects. Collectively, these findings suggest a major role for TEAD1 as an effector of the Hippo pathway during cerebellar development.

**Supplementary Information:**

The online version contains supplementary material available at 10.1007/s00429-025-02903-x.

## Introduction

The cerebellum is a hindbrain-derived structure with crucial roles for coordination of motor output, emotional processing and other sensorimotor behaviours (Manto et al. 2012; Amore et al. 2021). Abnormal cerebellar development underlies a range of disorders, including ataxias, autism spectrum disorder and attention deficit hyperactivity disorder (D'Arrigo et al. 2021; van der Heijden et al. 2021; Stoodley 2016). The cerebellum is also prone to the development of paediatric and adult cancers (Packer et al. 1999; Bhattacharya et al. 2020). Therefore, understanding the mechanisms by which cerebellar development is regulated is crucial to developing better treatments for these disorders/diseases.

Cerebellar development is a protracted process, beginning around the middle of gestation and ending around the third postnatal week in mice, with comparable timelines for human cerebellar development (Iskusnykh and Chizhikov 2022; Leto et al. 2016). The development of the cerebellum involves the generation of a multitude of developmentally distinct cell types, comprising various GABAergic and glutamatergic neurons, as well as non-neuronal cells such as Bergmann glia (BG) (Consalez et al. 2020; Consalez and Hawkes 2012; Araujo et al. 2019; Yamada and Watanabe 2002). Glutamatergic cerebellar granule neurons are the most populous neuronal cell type within the entire brain (Lee et al. 2009). Developmentally, these cells arise from a highly proliferative portion of the neuroepithelium known as the rhombic lip (Consalez et al. 2020). In mice, granule neuron development begins around embryonic day (E) 12 and results in the tangential migration of cerebellar granule neuron progenitors (CGNPs) to the nascent cerebellar neuroepithelium to populate an area known as the external granular layer (EGL). This is a transient secondary germinal zone, where CGNPs undergo a proliferative expansion prior to their differentiation and subsequent inwards migration to form granule neurons within the internal granular layer (IGL). This process concludes by the end of the 3rd postnatal week (Consalez et al. 2020; Chizhikov and Millen 2003). Other neuronal subtypes, including neurons of the deep cerebellar nuclei, as well as unipolar brush cells, are also derived from progenitors within the rhombic lip (Machold et al. 2011; Englund et al. 2006).

GABAergic inhibitory neurons, which include Purkinje neurons (PNs), basket and stellate cells and several other molecular layer and granule layer interneurons, as well as glial progenitors, derive from a separate pool of precursors within the embryonic neuroepithelium (Leto et al. 2012, 2008; Haldipur et al. 2018). These precursors derive from the ventricular zone adjacent to the rhombic lip between E10.5 and E13.5 (Luca et al. 2015) and follow a distinct trajectory to those cells derived from the rhombic lip (Chizhikov and Millen 2003). PNs are generated at ~ E11 in mice and migrate through the cerebellar anlage to form the nascent Purkinje cell layer (PCL) below the EGL, where they then secrete ligands crucial for the expansion of CGNPs (Luca et al. 2015; Wang et al. 2022). Similarly, radial glial progenitors (which include Bergmann glia and astrocytes) are specified at E14 in the ventricular zone, commence migration up to the first week of postnatal life, and subsequently begin to elaborate radial (Bergmann) fibres, which facilitate both the outgrowth of PN dendrites and the inwards migration of immature granule neurons from the EGL to the IGL (Consalez et al. 2020; Yamada and Watanabe 2002).

How are these complex populations of neurons generated spatially and temporally to form the mature cerebellum? Research has implicated numerous signalling pathways as being relevant to this process. For example, Shh signalling is crucial for the expansion of CGNPs in the EGL (Consalez et al. 2020), as well as regulating the proliferation of PAX2^+^ interneuron progenitors (Luca et al. 2015). Additionally, pathways such as WNT and Notch have also been shown to play similar roles in the development of the cerebellum, particularly in CGNP expansion within the EGL (Wang and Liu 2019). Another pathway critical for nervous system development is the Hippo pathway (Terry and Kim 2022). Hippo signalling involves a cascade of signalling molecules that ultimately drive transcription of genes associated with pro-proliferation, anti-apoptosis and the regulation of organ growth (Sahu and Mondal 2021; Misra and Irvine 2018). This cascade of molecules culminates within the nucleus, with the YAP/TAZ co-factors binding to the TEAD family of transcription factors. This complex then binds DNA and drives transcription of canonical pro-proliferative genes such as *Cyr61/Ccn1* and *Ctgf/Ccn2* (Malik et al. 2015; Mukhtar et al. 2020). The TEAD family have been shown to be important in a variety of developmental contexts, including cardiomyocyte proliferation (Wen et al. 2019), smooth muscle development (Wen et al. 2019; Osman et al. 2019; Kimura et al. 2020), skeletal muscle formation (Ribas et al. 2011; Sun et al. 2017; Feng et al. 2019; Honda et al. 2019) and generation of the pancreas (Cebola et al. 2015; Currey et al. 2021). Within the central nervous system, the TEAD family have been reported to contribute to neural tube closure (Kaneko et al. 2007), neural crest expansion (Gee et al. 2011) and neural stem cell biology within the cerebral cortex (Mukhtar et al. 2020; Currey et al. 2021). Importantly, distinct regulation of YAP/TAZ via Hippo-dependent and Hippo-independent mechanisms have recently begun to be elucidated (Kwon et al. 2022; Shreberk-Shaked and Oren 2019; Rausch and Hansen 2020). Further, YAP/TAZ have been demonstrated to have roles outside of the nucleus (Bui et al. 2016), suggesting a crucial role for Hippo signalling and its components in a variety of contexts.

Is Hippo signalling important for cerebellar development? A recent study revealed that cerebellar morphogenesis was disturbed in the absence of *Yap/Taz* (Hughes et al. 2020). However, whether this reflects Hippo-dependent or -independent pathways and which of the four TEAD factors may be engaged remains unclear. To investigate how Hippo-dependent signalling regulates cerebellar development, we studied the expression of TEAD1 within the postnatal cerebellum and assessed the phenotypic consequences of *Tead1* ablation on cerebellar morphogenesis. Our studies reveal that TEAD1 is expressed by CGNPs within the postnatal mouse cerebellum, as well as by glial progenitor cells and their progeny, including BG and astrocytes. Moreover, the conditional ablation of *Tead1* from neural stem cells, via a *Sox1*-Cre deleter, culminated in a range of both cell- and non-cell-autonomous deficits within the cerebellum. These phenotypes are reminiscent of the phenotype of mice lacking *Yap/Taz* (Hughes et al. 2020), suggesting that Hippo signalling regulates cerebellar development in part through a YAP/TAZ-TEAD1 transcriptional program.

## Materials & methods

### Animals

Animals used in this study were bred at The University of Queensland under approval from the Institutional Animal Ethics Committee (2023/AE000007). For the expression analyses wild-type C57BL/6 J mice were used. To study the role of *Tead1* in cerebellar development, a conditional allele was used; *Tead1*^*fl/fl*^ (*Tead1*^*tm1a(KOMP)Wtsi*^), which was generated by the Knockout Mouse Project (KOMP) consortium using embryonic stem cells (Collins et al. 2007). All embryonic stem cell injections, chimeric and germline mouse generation was performed at UC Davis KOMP Repository. Prior to use in our studies, the *lacZ-neo* cassette was removed via Flp-mediated deletion, as previously described (Skarnes et al. 2011). This conditional line was crossed with a *Sox1*^*Cre*^ line (Takashima et al. 2007), enabling the ablation of *Tead1* from neural stem cells within the nervous system from approximately E8. This generated *Tead1*^*fl/*+^*; Sox1*^*Cre/*+^ progeny. These animals were crossed with *Tead1*^*flfl*^ mice to generate control (*Tead1*^*fl/fl*^; *Sox1*^+*/*+^ or *Tead1*^*fl/*+^; *Sox1*^+*/*+^; hereafter referred to as controls), heterozygous (*Tead1*^*fl/*+^*; Sox1*^*Cre/*+^; hereafter referred to as cHet mice) or homozygous (*Tead1*^*fl/fl*^*; Sox1*^*Cre/*+^*;* hereafter referred to as cKO mice) animals. All experiments were performed according to the Australian Code of Practice for the Care and Use of Animals for Scientific Purposes. Pregnant females were acquired by placing male and female mice together overnight. The next day, females were inspected for the presence of a vaginal plug. The day of birth was designated as postnatal day (P) 0. Mice were housed in Optimice IVC caging, with double HEPA filter and built in ventilation. Food and water were available ad libitum. Animals at P0, P5, P20 and P56 were used in this study. Mice were genotyped by PCR; primers are available on request.

### Immunohistochemistry

Postnatal pups were transcardially perfused (1X phosphate buffered saline, followed by 4% paraformaldehyde (PFA)) and postfixed in 4% PFA at 4 °C as previously described (Oishi et al. 2016). Brains were removed from the skull and processed in a Leica ASP300S Pathcentre prior to embedding in paraffin. Brains were embedded sagittally and sectioned at 12 µm (Leica RM2245 Microtome). Mid-sagittal sections were mounted on Superfrost + slides and dried in a 37 °C oven before heat-mediated antigen retrieval was performed in 10 mM sodium citrate solution (90 °C for 10 min). Fluorescence immunohistochemistry (IF) was then performed as previously described (Piper et al. 2010). Briefly, sections were incubated overnight with primary antibodies against target proteins (Table 1). The following day, sections were rinsed in phosphate buffered saline (PBS) and then incubated with the relevant secondary antibodies for 3 h at room temperature. A list of all antibodies used is listed in Table 1. Sections were rinsed in 0.9% saline and counterstained with 4’, 6-diamidino-2-phenylindole (DAPI) and mounted in fluorescence mounting media (DAKO).

**Table 1:** Details of all antibodies used in this study for IF analyses

ANTIBODY	SPECIES	COMPANY	CATALOG #	DILUTION
TEAD1	Rabbit	Abcam	EPR3967(2)	1/800
KI67	Mouse	BD Biosciences	550,609	1/1000
PAX6	Rabbit	BioLegend	901,301	1/400
GFAP	Mouse	MilliporeSigma	MAB360	1/500
Parvalbumin	Mouse	MilliporeSigma	P3088	1/400
Calbindin	Mouse	MilliporeSigma	C9848	1/300
SOX2	Rat	eBioscience	14–9811-82	1/500
TBR2	Sheep	Thermofisher	PA5-47,818	1/400
NFIX	Mouse	Sigma-Aldrich	SAB1401263	1/400
SECONDARY ANTIBODIES & DAPI				
Mouse α 647	Donkey	Jackson ImmunoResearch	715–605-150	1/1000
Rabbit α 555	Donkey	Jackson ImmunoResearch	711–165-152	1/1000
Rat α 555	Goat	Thermofisher	A-21434	1/1000
Goat α 594	Donkey	Abcam	ab150132	1/1000
DAPI	N/A	Thermofisher	D1306	1/500

### Histological staining

Postnatal pups were transcardially perfused (1X PBS, followed by 4% PFA), postfixed in 4% PFA at 4 °C and processed to paraffin as described above. Brains were embedded sagittally and sectioned at 12 µm (Leica RM2245 Microtome) and mid-sagittal sections were stained with Haematoxylin and Eosin (H&E) using standard protocols (Piper et al. 2011). For all H&E-stained images, sections were imaged using a brightfield slide scanner (Aperio XT Brightfield) at 40 × magnification. Images were captured using ScanScope XT, and post-processed using Aperio ImageScope ×64. Images were exported as tiffs and were quantified in blinded conditions.

### Imaging and cell counts

To analyse the colocalisation of TEAD1 with cell type-specific proteins, images were acquired on a Zeiss LSM900 Airyscan 2 confocal microscope at 20 x (0.8 NA) magnification. Data were acquired as Airyscan Multiplex-4Y (MPLX4Y) images to improve signal-to-noise and spatial resolution compared with standard laser scanning confocal microscopy (Huff 2019, 2015). Variable beam splitters restricted the emission wavelengths captured to prevent fluorescence bleed through between the DAPI (detection wavelength 400–494 nm), AlexaFluor 555 (detection wavelength 529–628 nm), AlexaFluor 594 (detection wavelength 562–630 nm) and AlexaFluor 647 channels (detection wavelength 630–700 nm). The MPLX4Y images were acquired as 8 μm tiled z-stacks (0.34 μm z-step), which were stitched and rendered as maximum intensity projections using ZEN Blue software. To quantify cell number and morphology within the whole cerebellum, 8 μm tiled z-stacks (0.49 μm z-step) of control, cHet and cKO cerebella were captured at 40 × magnification (0.95 NA) using a Zeiss Axioscan Z1 slide scanner with a Colibri 7-LED light source, pentaband emission filter set (112 MBP) and Hamamatsu Orca Flash 4 sCMOS camera. The tiled z-stacks were stitched and rendered as maximum intensity projections using ZEN Blue software.

In all cases except molecular layer (ML) width, cross-sectional area quantification, TEAD1/marker co-localisation, and Ki67 and PN cell number, the StarDist plugin for ImageJ (Rueden et al. 2017) was used to automate the cell count process and to minimise bias. Default settings (available from StarDist’s documentation) were used for all cell counts, except for the number of tiles – this was set to 40 to accommodate whole section counts for the largest sections to be performed. Following StarDist, images were thresholded (1 and 65,535) and a mask was created. The number of cells was then counted using Analyse Particles. Cell count validity was verified by performing automated and manual blinded counts on sections of lobes and confirming similar numbers were reached by researcher and StarDist as previously described (Schmidt et al. 2018; Weigert et al. 2020; MaS and Uwe 2020; Fraser et al. 2017). For PAX6 cell counts, the IGL was traced and removed from all images, as this area was too dense for reliable quantification via StarDist. For PCL-localised SOX2^+^ cell number, the PCL was traced using the positioning of PCL soma (large acellular areas along the cell-dense IGL) for Lobes 6/7. SOX2 cell number was then quantified using StarDist as described above.

For molecular layer width, the straight-line tool in Fiji was used to measure the distance between the PCL and pial surface (3 lobes/brain). For cross-sectional area quantifications, H&E images were loaded in Fiji and scale was set for the image. The outermost edge of each section was continuously traced using the polygon tool, and a measure of the total cerebellar area was obtained. All quantifications (manual or automated) were performed on whole sections from three biological replicates for control, cHet and cKO cerebella.

### Volumetric analyses

The Australian Mouse Brain Project Atlas for the cerebellum was downloaded (https://imaging.org.au/AMBMC/) and registered to each of the samples B0 images (Ullmann et al. 2012). This was done using FSL and ANTS software packages (Supp. Table S1). Rigid and deformation warping was optimised and conducted using these packages. ITKSnap software packages (Supp. Table S1) were used to manually adjust the cerebellum atlas region template to matched anatomical areas. Volumetric analyses (students *t*-test with Holm-Sidak correction) were then conducted to determine whole structure volume, and individual region volumes. For all volumetric analyses, n = 8 Ctrl and n = 8 cKO age- and sex-matched adult (P56) mice were used, with an approximately even split of sexes.

### Statistical analyses

All cell count data was validated using a one-way ANOVA with multiple comparisons of control, cHet and cKO data unless specified otherwise. All data are presented as mean ± SEM. Three biological replicates were used for all IF quantifications.

## Results

### Expression of TEAD1 within the postnatal cerebellum

**Fig. 1:**
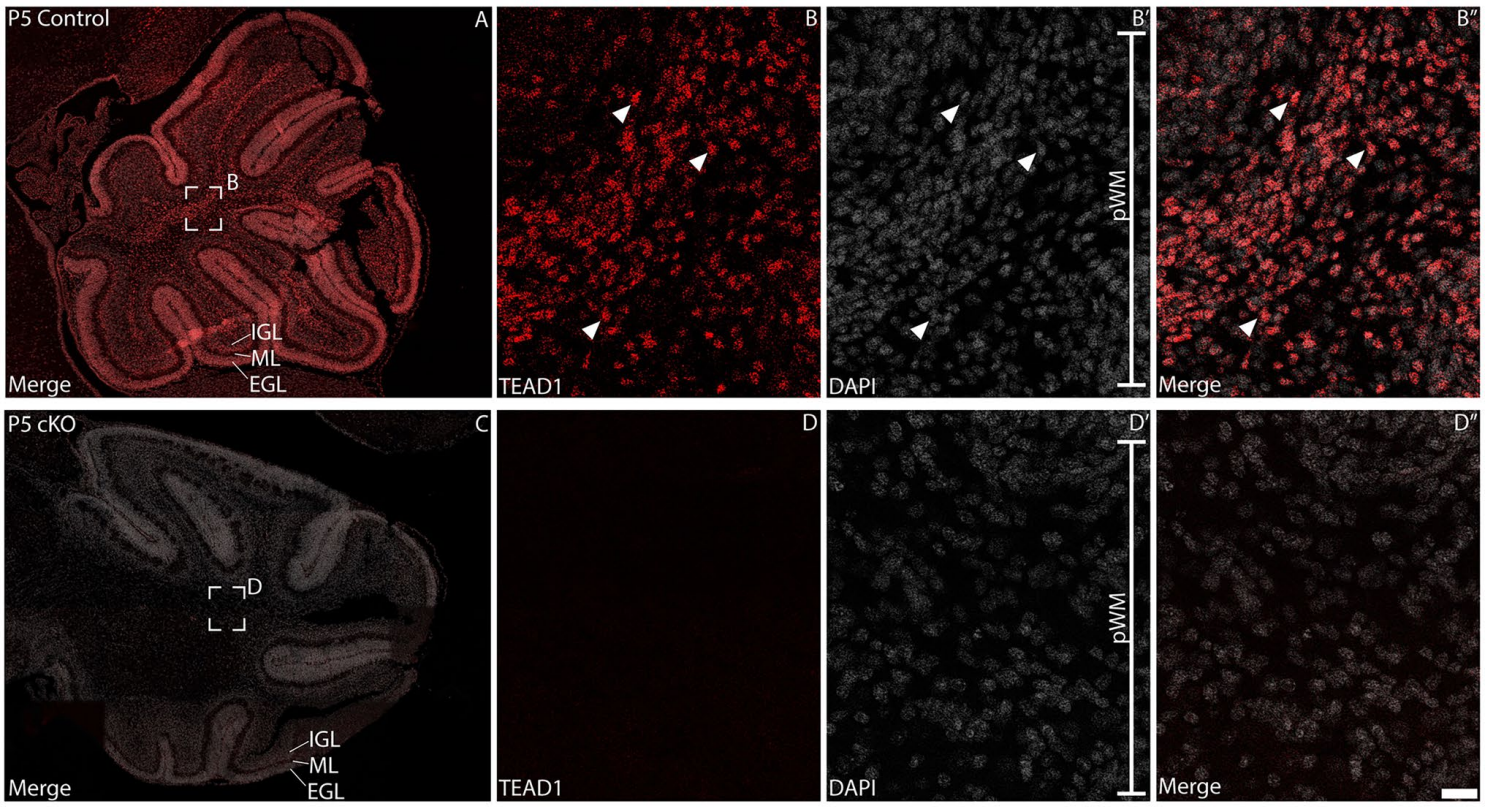
TEAD1 is expressed within the postnatal mouse cerebellum. Sagittal sections taken from control (**A-B**) and cKO (**C-D**) P5 cerebella at the level of the vermis, revealing expression of the transcription factor TEAD1 (red). Nuclei were labelled with DAPI (grey). Boxed regions in A and C are shown at higher magnification in **B** and **D**, respectively. In the control, TEAD1 expression was evident within nuclei in the internal granular layer/white matter (IGL/WM; arrowheads in **B-B**”). However, in the cKO cerebellum, no immunoreactivity was observed (D-D”). Scale bar (in D”) represents 165 µm for A and C and 20 µm for B-B”, **D-D**”

TEAD1 expression has been reported within the cerebellum of several different mammalian species at the transcriptomic level, including the mouse (Supp. Fig. S1) (Brawand et al. 2011), however the cell type-specific expression patterns of TEAD1 protein within the cerebellum remains unclear. To address this, we first performed immunofluorescence staining on sagittal sections of the P5 mouse cerebellum using a TEAD1-specific antibody. This revealed clear and specific nuclear staining of TEAD1 within CGNPs and by cells within the PCL and white matter at this age (Fig. 1A-B). Importantly, analysis of P5 cKO tissue revealed that no immunopositive cells were present (Fig. 1C-D). This is consistent with recent findings from our laboratory detailing the expression of TEAD1 within the developing telencephalon (Pelenyi et al. 2024). These data are important for three reasons. Firstly, the data reveal that our conditional knockout strategy is effective. Secondly, they reveal that our TEAD1 antibody is specific and does not demonstrate cross-reactivity with related family members. Finally, they also reveal that TEAD1 is expressed widely within the postnatal cerebellum. To investigate this further, we next used co-immunofluorescence staining to map the cell type specific expression of TEAD1 within the postnatal cerebellum.

### Proliferative cells within the postnatal EGL express TEAD1

**Fig. 2:**
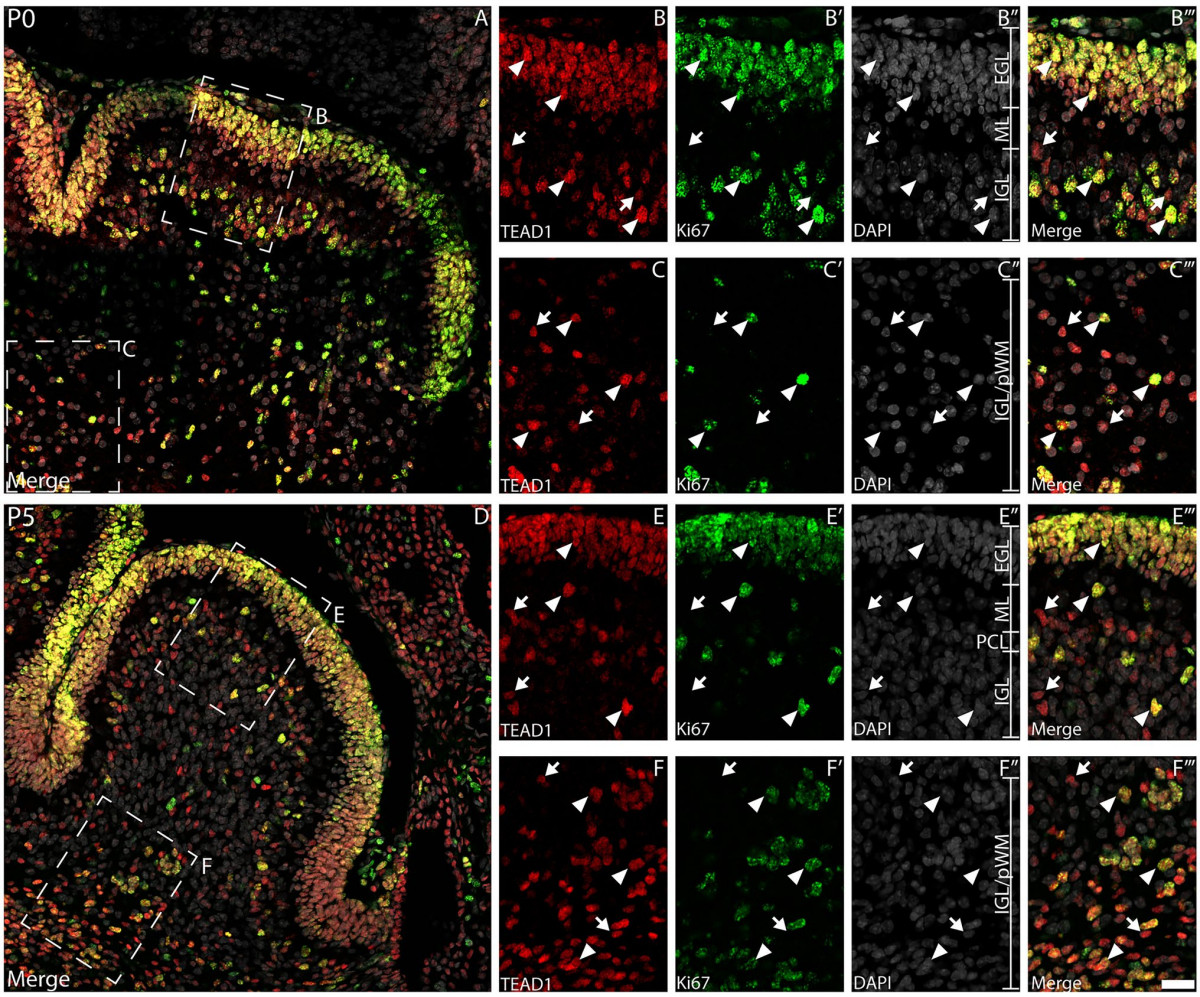
Proliferative cells within the developing cerebellum express TEAD1. Mid-sagittal sections taken from wild-type cerebella at P0 (**A-C**”’) and P5 (**D-F**”’), revealing the expression of TEAD1 (red) and Ki67 (green). Nuclei were labelled with DAPI (grey). Low magnification images in A and D revealed broad colocalisation of TEAD1 and Ki67 across both ages. At P0, expression of TEAD1 colocalised with Ki-67 in both the EGL/IGL (**B-B**’”) and the presumptive white matter (pWM; **C-C**”′) (arrowheads). However, in both the EGL and pWM, there were also non-proliferative TEAD1^+^/Ki67^−^ cells present (arrows). Similarly, at P5, TEAD1^+^/Ki67^+^ populations were identified within the EGL/IGL (**E-E**’”) and pWM (**F-F**’”) (arrowheads), as were some TEAD1^+^/Ki67^−^ cells (arrows). Scale bar in (**F**’”) represents 30 µm for A & D and 20 µm for all other panels

**Fig. 3:**
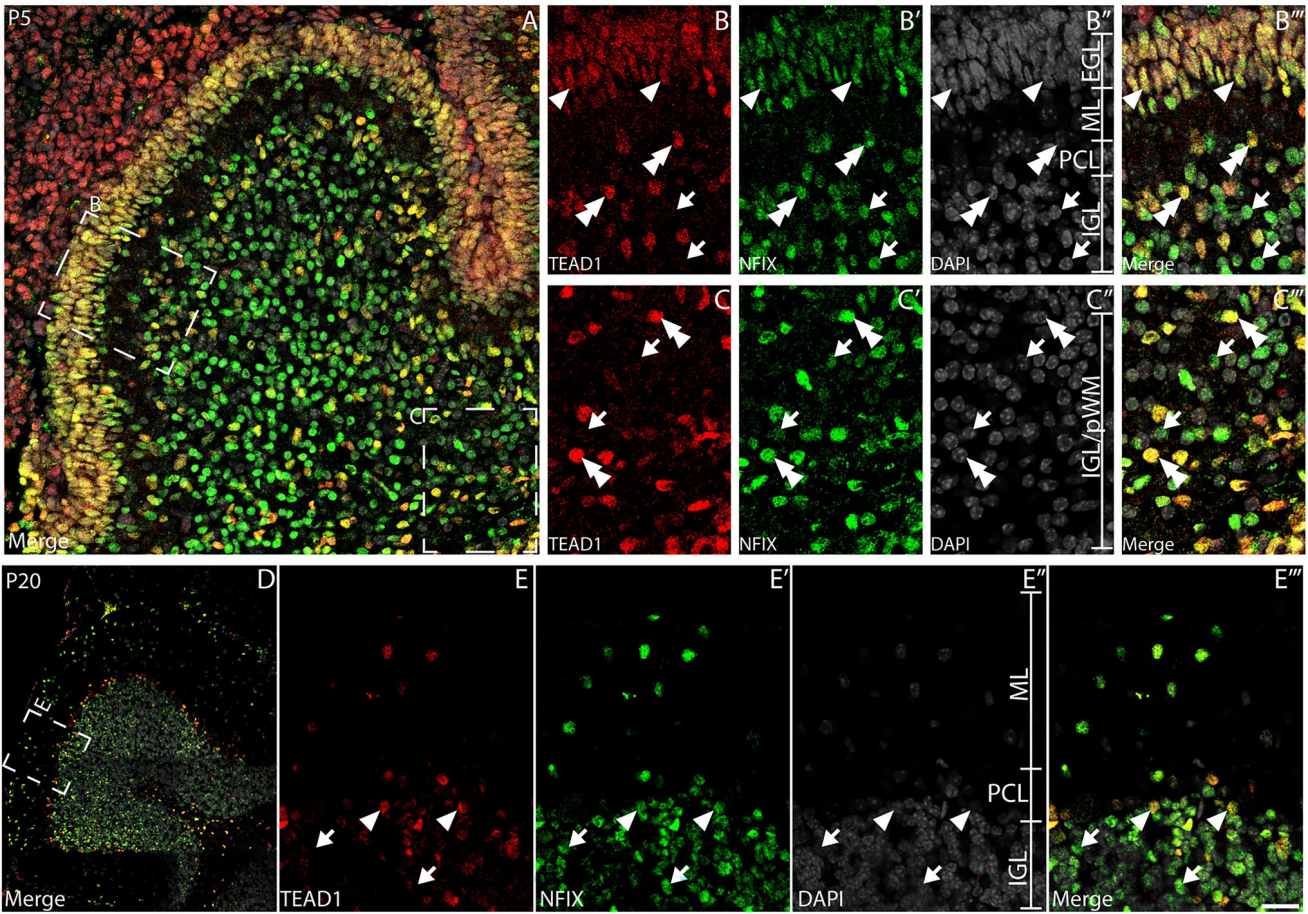
Cerebellar granule neuron precursors, but not mature granule neurons, express TEAD1. Mid-sagittal sections of wild-type cerebella at P5 (**A-C**’”) and P20 (**D-G**) demonstrating expression of TEAD1 (red) and NFIX (green). Nuclei were labelled with DAPI (grey). At P5, expression of TEAD1 was coincident with NFIX within the EGL (arrowheads) and within sub-populations of cells outside the EGL (double arrowheads). Cells were also evident within the nascent IGL and pWM which expressed NFIX, but not TEAD1 (arrows). Conversely, at P20, expression of TEAD1 was coincident with NFIX only within the PCL (arrowheads). Cells within the IGL (arrows) and ML (double arrows) which were immunopositive for NFIX did not express TEAD1, suggesting that TEAD1 is not expressed in mature granule neurons. Scale bar in (**E**’”) represents 30 µm for A, 50 µm for D, 20 µm for **B-C**’” and 10 µm for **E-E**’”

Our preliminary data (Fig. 1A) suggested that TEAD1 was expressed by CGNPs within the EGL at P5. CGNPs divide rapidly during postnatal development—as such, we used the expression of the proliferative marker, Ki67, to verify if these cells express TEAD1. Dual labelling with antibodies against Ki67 revealed that proliferating CGNPs within the P0 and P5 EGL expressed TEAD1 (Fig. 2A, D). Interestingly, this analysis also revealed cells within the PCL, the emerging IGL (Fig. 2B, E) and the white matter (Fig. 2C, F) that expressed TEAD1; some of these cells were proliferating (Ki67 positive), whereas others were not (Ki67 negative). To begin identifying these additional TEAD1-expressing populations, we performed dual labelling with antibodies against the transcription factor NFIX, which we have previously shown is expressed by CGNPs within the P5 mouse cerebellum, and by immature and mature granule neurons within the IGL (Fraser et al. 2017). As expected, at P5 a high percentage of CGNPs co-expressed both NFIX and TEAD1 within the EGL (Fig. 3A-B’”; 137 out of 150 (91.3%) CGNPs expressed both factors). Interestingly, at P5 there was evidence for some cells expressing both NFIX and TEAD1 within the IGL, however, most cells expressed NFIX, but not TEAD1. This suggests that CGNP-derived granule neurons do not express TEAD1. Analysis of P20 cerebellar tissue confirmed this, revealing that NFIX-expressing granule neurons within the IGL do not express TEAD1 (Fig. 3D-E’”). Collectively, these data suggest that CGNPs express TEAD1, but that the expression of this transcriptional regulator is rapidly downregulated as these cells differentiate into granule neurons.

### Purkinje neurons do not express TEAD1

**Fig. 4:**
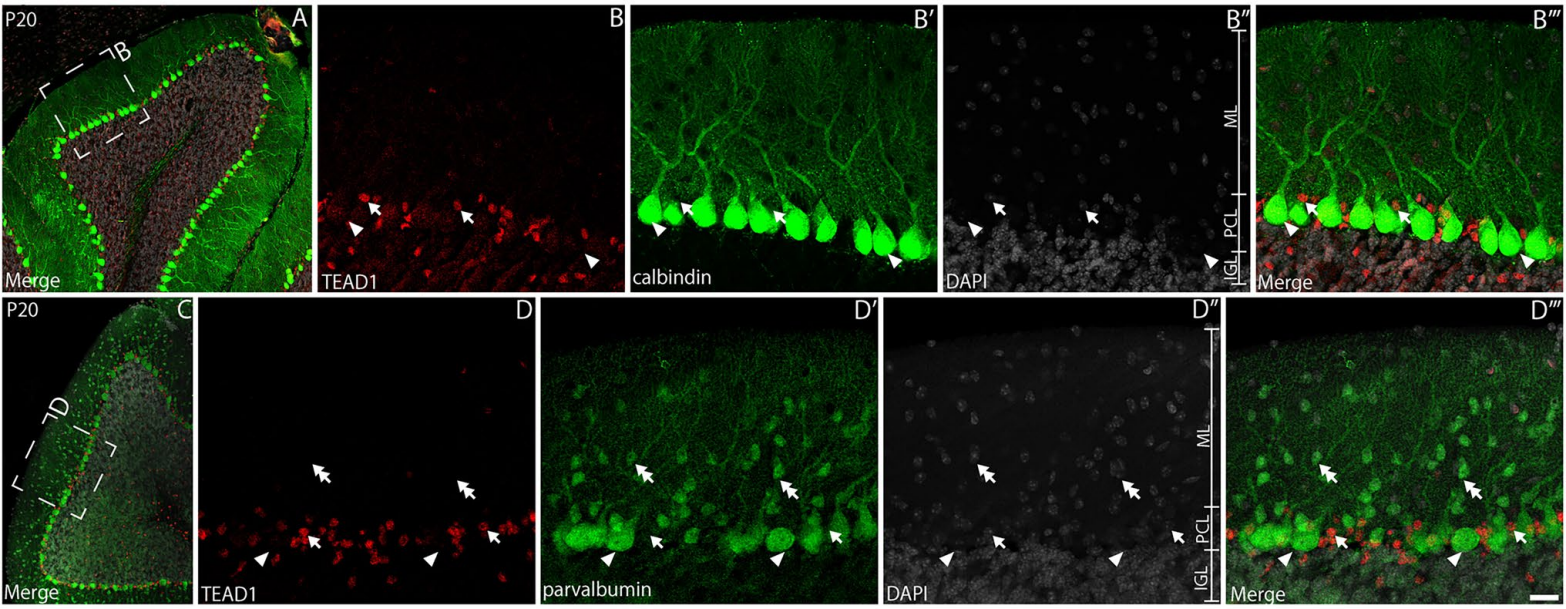
Mature Purkinje neurons and molecular layer interneurons do not express TEAD1. Mid-sagittal sections taken from wild-type cerebella at P20, revealing the expression of TEAD1 (red) and calbindin (**A-B’”**) or parvalbumin (**C–D’”**) in green. Nuclei were labelled with DAPI (grey). (**A-B’”**) High magnification images revealed that calbindin-expressing PNs did not express TEADs in the PCL (arrowheads). Numerous cells in the PCL were shown to express TEAD1, but not calbindin (arrows). (**C–D’”**) Similarly, expression of another PN marker, parvalbumin, revealed that PNs do not express TEAD1 at this age (arrowheads). Furthermore, interneurons of the ML (basket and stellate cells) were not immunoreactive for TEAD1 (double arrows). Small nuclei within the PCL were, however, immunoreactive for TEAD1 (arrows). Scale bar in (**D**’”) represents 100 µm for A and C and 20 µm for all other panels

Despite our data indicating that mature granule neurons do not express TEAD1, we did identify a population of cells between the IGL and the molecular layer that expressed this factor (Fig. 3D-E). The PCL lies between the molecular layer and the IGL, and houses the soma of PNs and BG. To determine which of these cells express TEAD1, we first performed co-immunofluorescence staining for TEAD1 and calbindin, a marker for PNs (Wierzba-Bobrowicz et al. 2011). Analysis at P20 revealed a population of cells with small nuclei expressing TEAD1 within the PCL, however, these cells were not immunoreactive for calbindin, but rather were interspersed between the large soma of calbindin-expressing cells (Fig. 4A-B’”). A similar finding was found when we used a second marker for PNs, parvalbumin (Fig. 4C-D’”) (Bastianelli 2003). These data suggests that PNs do not express TEAD1. Furthermore, parvalbumin also labels interneurons within the molecular layer. We did not observe co-expression of TEAD1 by these cells (Fig. 4C-D’”), suggesting that, like PNs, basket and stellate interneurons do not express TEAD1.

### Glial progenitor cells, Bergmann glia and astrocytes express TEAD1 within the postnatal cerebellum

**Fig. 5:**
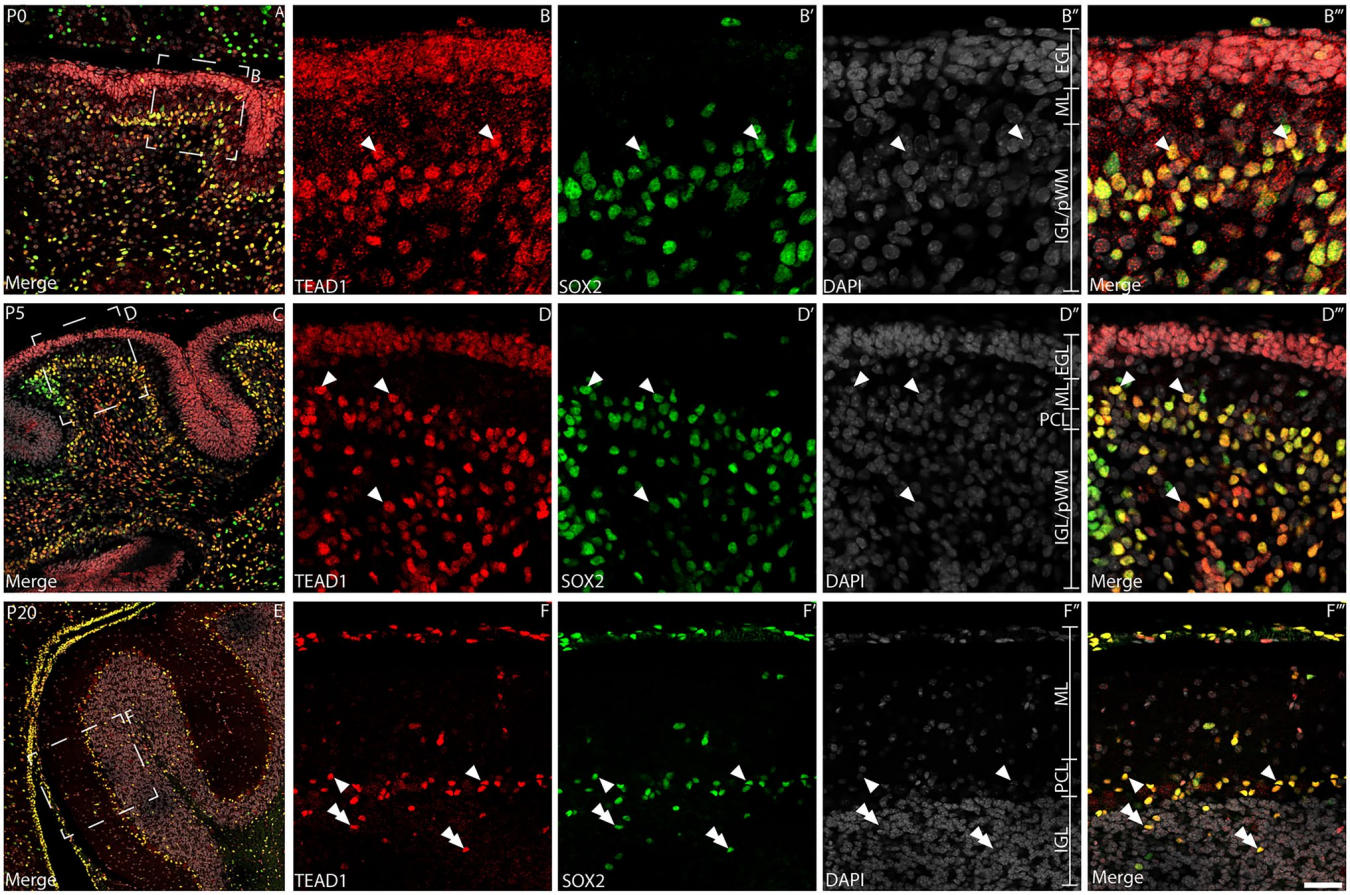
Glial lineages express TEAD1. Mid-sagittal sections of wild-type cerebella at P0 (**A-B**’’’), P5 (**C-D**’’’) and P20 (**E–F**’’’), demonstrating expression of TEAD1 (red) and SOX2 (green). Nuclei were labelled with DAPI (grey). At P0, expression of TEAD1 was evident within the EGL, while TEAD1/SOX2 co-expression was observed by cells within the IGL/pWM (arrowheads in B-B’’’). At P5, expression of TEAD1 was seen within nuclei located in the EGL and IGL – however, colocalisation with SOX2 was only evident within the IGL (arrowheads in **D-D**’’’). At P20, some scattered cells within the ML and cells within the PCL expressed both markers (arrowheads in **F-F**’’’). There were also cells within the IGL positive for both markers (double arrowheads). Scale bar (in **F**’’’) represents 50 µm for A, C and E and 20 µm for all other panels

**Fig. 6:**
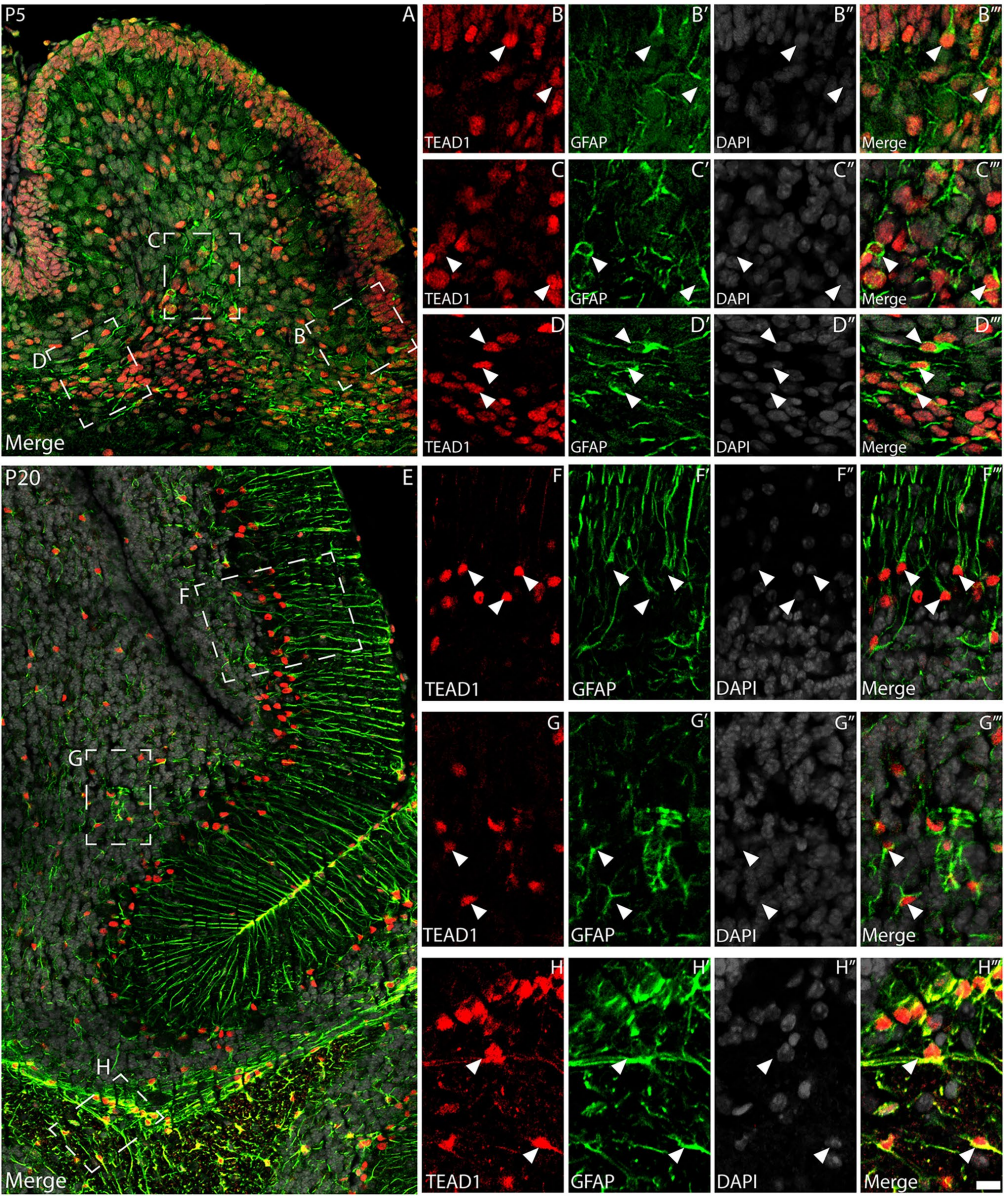
Astrocytes express TEAD1 throughout development. Mid-sagittal sections taken from wild-type cerebella at P5 (**A-D**’”) and P20 (**E-H**’”), revealing the expression of TEAD1 (red) and GFAP (green). Nuclei were labelled with DAPI (grey). (A-D’”) Low magnification image in A reveals broad co-localisation of TEAD1 and GFAP within the developing cerebellum. Higher magnification images reveal many TEAD1-expressing nuclei surrounded by GFAP-expressing fibres within the nascent PCL/IGL (**B**), deep IGL (**C**) and pWM (**D**) (arrowheads), indicative of TEAD1 expression within various astrocytic progenitor populations (including BG/radial glia, velate protoplasmic astrocytes and fibrous astrocytes). (**E-H**’”). Low magnification image in E reveals colocalisation of TEAD1 and GFAP across the mature cerebellum. Higher magnification images reveal numerous TEAD1-expressing nuclei (arrowheads) surrounded by GFAP-expressing fibres within the PCL (BG; **F**), the IGL (velate protoplasmic astrocytes; **G**) and the pWM (fibrous astrocytes; **H**). Scale bar in (**H**’”) represents 20 µm for A and 25 µm for E and 10 µm for all zoom panels

Given the expression of TEAD1 within the PCL and IGL, but not by PNs or granule neurons, we posited that glial cells may be responsible for these expression patterns. To investigate this, we co-stained P0, P5 and P20 cerebellar sections with antibodies against TEAD1 and SOX2, a marker expressed by glial progenitor cells and mature glia within the cerebellum (Cerrato et al. 2018). Consistent with our earlier findings, we observed expression of TEAD1 by CGNPs within the EGL at P0 and P5 (Fig. 5A-D’”). Furthermore, within the emerging PCL and IGL at these ages, most TEAD1-expressing cells also expressed SOX2, demonstrating that glial progenitors and developing BG express TEAD1 during the genesis of the cerebellum. Within the mature P20 cerebellum, most BG within the PCL were immunoreactive for TEAD1 and SOX2 (148/150 colocalised cells, 98.7% colocalisation), as were astrocytes within the ML and IGL (Fig. 5E-F’”, 131/151 colocalised cells, 86.8% colocalisation). These findings were supported using another astrocytic marker, GFAP. At P5, developing TEAD1-expressing nuclei within the PCL, IGL and white matter were surrounded by GFAP-immunoreactive fibres (Fig. 6A-D’”). This pattern of expression was also clear at P20 with BG (Fig. 6F-F’”), velate protoplasmic astrocytes within the IGL (Fig. 6G-G’”), and fibrous astrocytes within the white matter (Fig. 6H-H’”), all exhibiting GFAP-immunopositive fibres surrounding TEAD1-expressing nuclei (Fig. 6E). Taken together, these data suggest that TEAD1 is expressed in developing and mature cerebellar BG and astrocytes, suggestive of a crucial role for *Tead1* in regulating these populations.

### Expression of TEAD1 by unipolar brush cells

Finally, we observed that a subset of cells within the IGL expressed TEAD1, but not GFAP. These cells could be unipolar brush cells, a population of glutamatergic interneurons found within the IGL. Like CGNPs, these cells are ultimately derived from progenitor cells of the rhombic lip (Kalinichenko and Okhotin 2005). Using the expression of TBR2 as a marker for these glutamatergic interneurons (Englund et al. 2006), we revealed that most unipolar brush cells within the IGL do express TEAD1 within the P20 cerebellum (Supp. Fig. S2A-D).

### Cerebellar development is impaired in the absence of *Tead1*

**Fig. 7:**
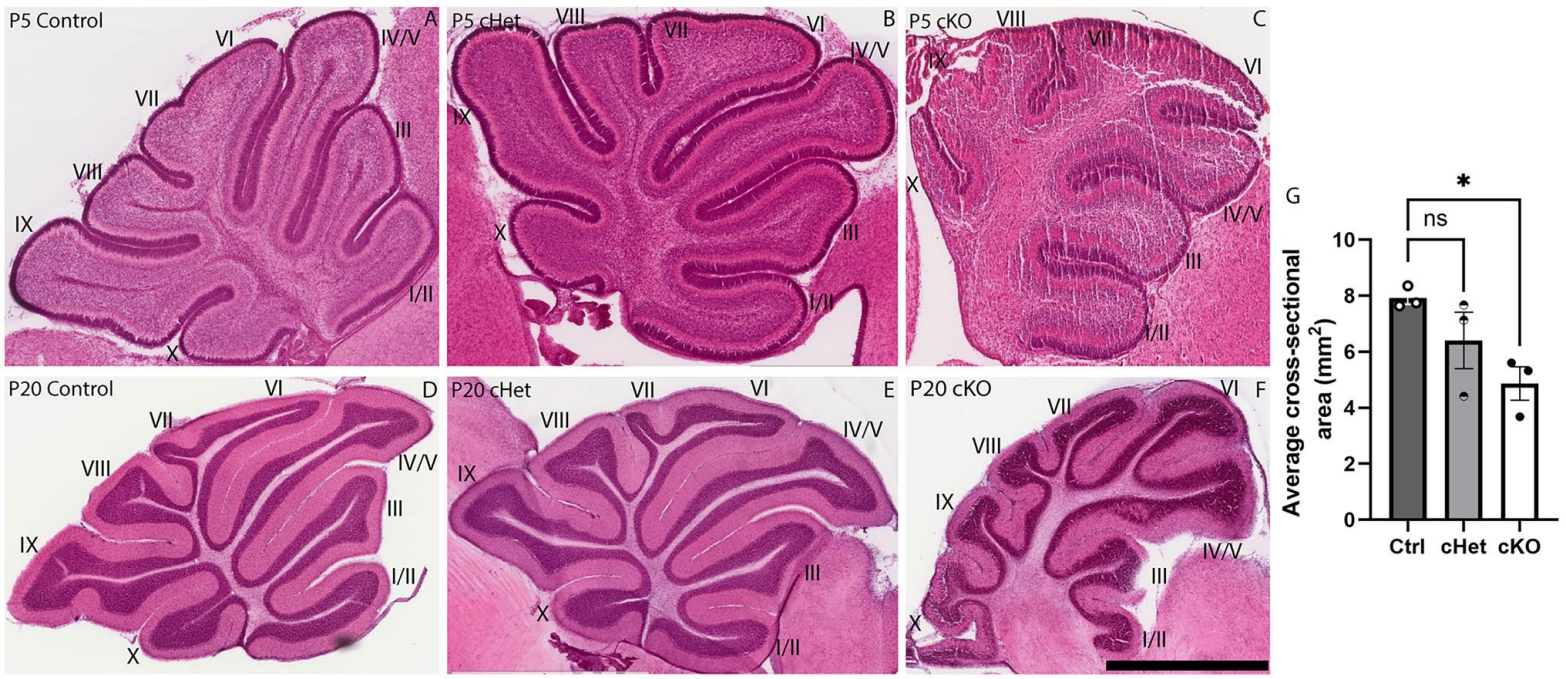
Cross-sectional area of the cerebellum is reduced in the absence of *Tead1*. Mid-sagittal sections of P5 (**A-C**) and P20 (**D-F**) cerebella, stained using Haematoxylin and Eosin. At P5, both control and cHet cerebella exhibited normal morphological development, with all folia present and correctly formed. cKO mice, however, exhibited less clear development of the folia, particularly within the posterior lobes (**A**-**C**). This phenotype was also evident at P20, with cKO mice exhibiting clear disturbances in morphogenesis of the cerebellum (**D**-**F**). Quantification of cross-sectional area revealed the cKO cerebellum was significantly smaller at this age (**G**). Scale bar in F represents 375 μm for A-C and 2 mm for **D-F**. ns, not significant; * = *p* < 0.05, one-way ANOVA

We next sought to assess the role of TEAD1 in cerebellar development. To do this, we conditionally deleted *Tead1* from neural stem cells within the developing murine nervous system using a *Sox1*^*Cre*^ deleter. This approach effectively ablated TEAD1 expression from the postnatal cerebellum (Fig. 1C-D). To confirm if morphological abnormalities were evident in the absence of *Tead1*, we performed Haematoxylin & Eosin (H&E) staining to visualise gross cerebellar morphology. Importantly, at P5, the cKO cerebellum exhibited morphological abnormalities, with the size of the cKO cerebellum being reduced, and the cerebellar folia being less well developed in comparison to the control (Fig. 7A-C). This phenotype was clearer at P20, with cKO cerebella exhibiting dysmorphic folia (Fig. 7D-F), as well as being significantly reduced in size (Fig. 7G). At P20, the midsagittal cross-sectional area of cHet mice was reduced, albeit not at a statistically significant level in comparison to the control (Fig. 7G). Unfortunately, we were unable to extend our analyses of the cerebellum of cKO mice beyond P20, as these mice died around this age. However, cHet mice survived into adulthood allowing us to determine whether the cerebellum of adult cHet mice exhibited alterations in size. To this end, we performed volumetric magnetic resonance imaging, which revealed that the size of the adult cHet cerebellum was significantly reduced in comparison to controls, as were a range of cerebellar sub-structures (Supp. Fig. S3A-D, Table S2). We recently reported a similar reduction in the size of the adult telencephalon and diencephalon in *Tead1* cHet mice in comparison to controls (Pelenyi et al. 2024), indicating that the reductions seen within the size of the cerebellum are not unique to this structure in this model. Collectively, these findings suggest that the loss of *Tead1* during development alters the trajectory of cerebellar development, culminating in reduced cerebellar size, and highlighting the crucial role *Tead1* plays in the development of this structure.

### Bergmann glial fibres are dysmorphic in the absence of *Tead1*

**Fig. 8:**
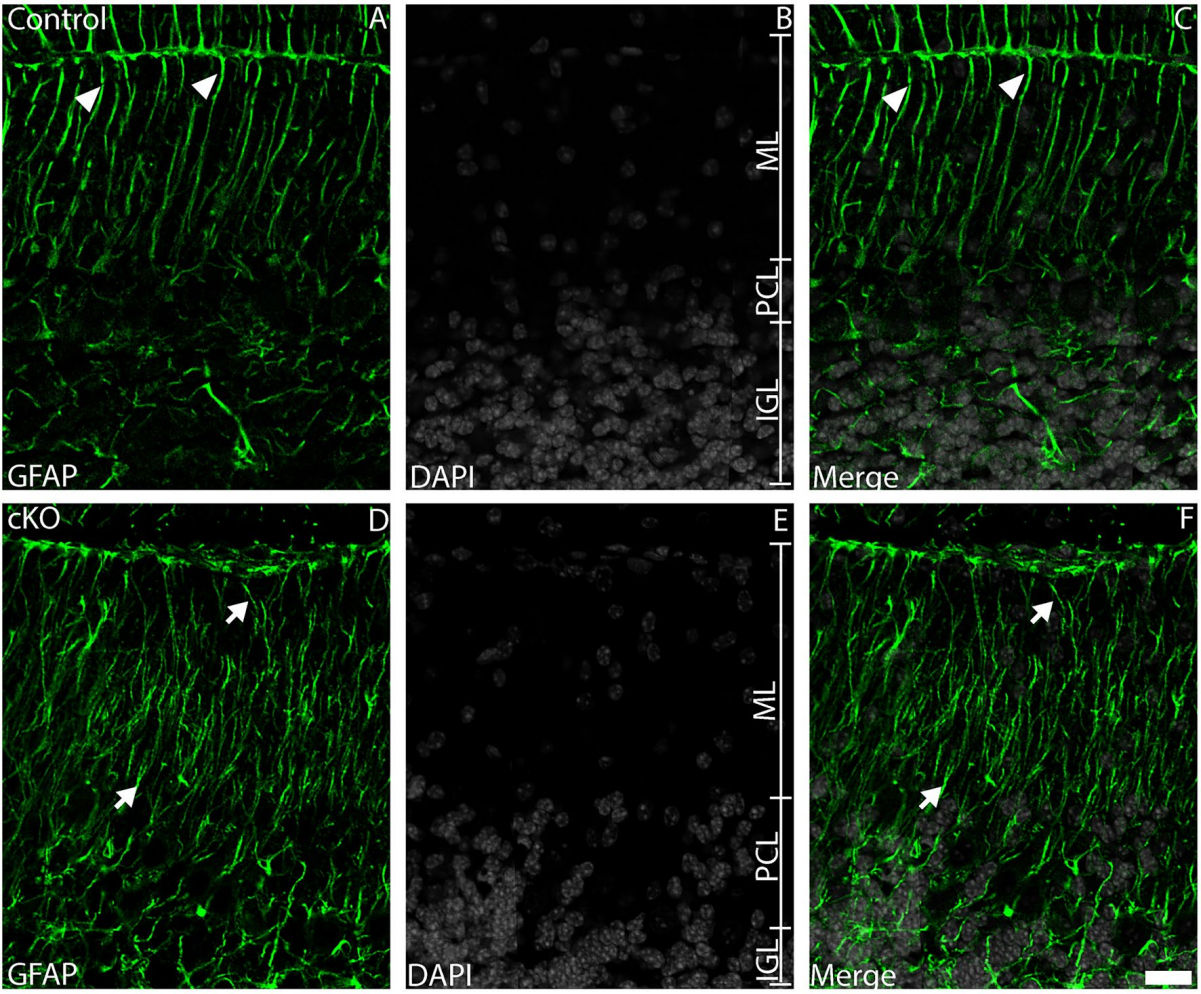
BG architecture is dysmorphic in the absence of *Tead1*. Mid-sagittal sections taken from cerebella at P20, revealing the expression of GFAP (green) in control (**A-C**) and cKO (**D-F**) mice. Nuclei were labelled with DAPI (grey). High magnification images reveal typical glial fibre architecture in control mice, with BG fibres extending straight up from the PCL to the pial surface (arrowheads, **A**-**C**). In the cKO however, BG fibres did not extend straight up, instead exhibiting tortuous and curved orientations (arrows, **D**-**F**). Scale bar (in F) represents 20 µm for all panels

Given the dysmorphic development exhibited by the cKO cerebellum, we wanted to investigate different cellular populations within these mice. BG have several crucial roles in the morphogenesis of the cerebellum (Cerrato et al. 2018), including acting as a scaffold for migrating granule neurons, and for facilitating PN dendrite arborisation. Is BG morphology altered in cKO mice? To investigate this, we analysed GFAP expression. At P20, the BG fibres within control cerebella extended from the PCL directly to the pial surface through the molecular layer (Fig. 8A-C). cKO mice, however, exhibited BG fibres which were more tortuous, as well as being shorter (Fig. 8D-F). Additionally, we also quantified SOX2^+^ cell number within the P20 control and cKO cerebellum. This analysis revealed no significant changes in the number of SOX2-expressing cells within the PCL of the mutant compared to the control (Supp. Fig. S4A-F, H). However, this analysis did reveal a significantly increased number of SOX2-expressing cells within the IGL of cKO mice (Supp. Fig. S4G). Collectively, these findings are indicative of cell-autonomous deficits within cerebellar glia in the absence of *Tead1*, consistent with previous studies on Hippo function during cerebellar development (Hughes et al. 2020)*.*

### Purkinje neurons exhibit dysmorphic development in the absence of *Tead1*

**Fig. 9:**
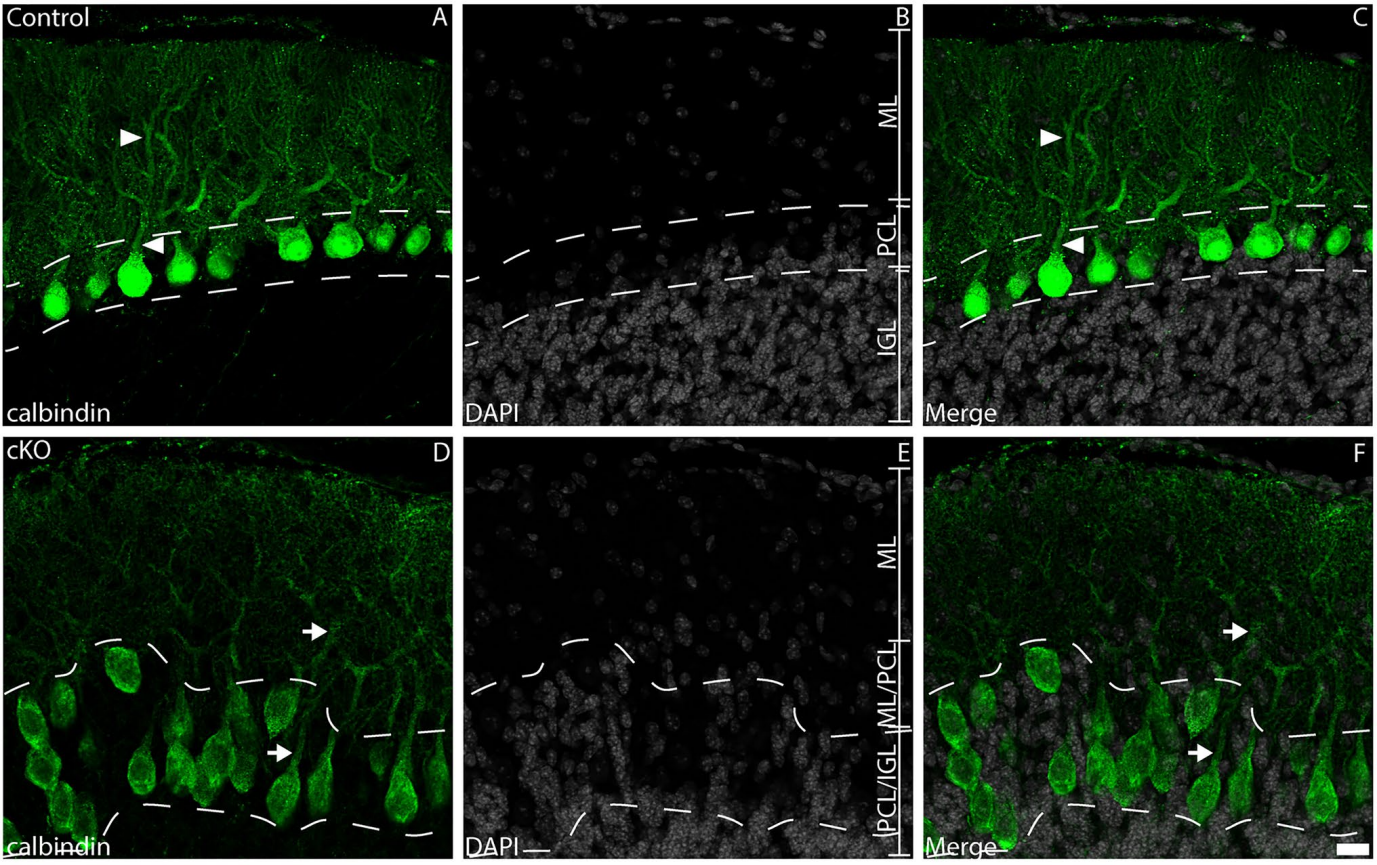
PN morphology and localisation is disturbed in the absence of *Tead1*. Mid-sagittal sections of control (**A-C**) and cKO (**D-F**) cerebella at P20, revealing the expression of calbindin (green); nuclei were labelled with DAPI (grey). In control cerebella, formation of the PCL monolayer (dotted lines demarcate the PCL) was evident. PNs also exhibited elaborate dendritic trees at this age (arrowheads). In comparison, cKO cerebella exhibited disturbances with PN monolayer formation (dotted lines). Additionally, PN dendritic architecture, while evident, appeared less well defined at this age (arrows). Scale bar (in F) represents 20 µm for all panels

BG regulate PN development (Bellamy 2006) – could the glial phenotype we observed contribute to non-cell-autonomous effects within PNs? To determine this, we analysed calbindin expression in P20 control and cKO mice. At P20, calbindin expression revealed a monolayer of PNs above the IGL in control samples. Moreover, these PNs possessed elaborate dendritic trees (Fig. 9A-C). In contrast, cKO mice exhibited a dysmorphic PCL, with no clear monolayer formation of PN soma compared with controls (Fig. 9D-F). There was no significant difference in overall PN soma number in the mutant compared to the control. Additionally, while cKO PNs did possess dendrites, these were less elaborate than that observed in controls. Analysis with a second PN marker, parvalbumin, supported these findings (Supp. Fig. S5A-F) In total, these findings suggest a key role for *Tead1* in regulating PN development in a cell-non-autonomous manner.

### *Tead1* is required for granule neuron migration

**Fig. 10:**
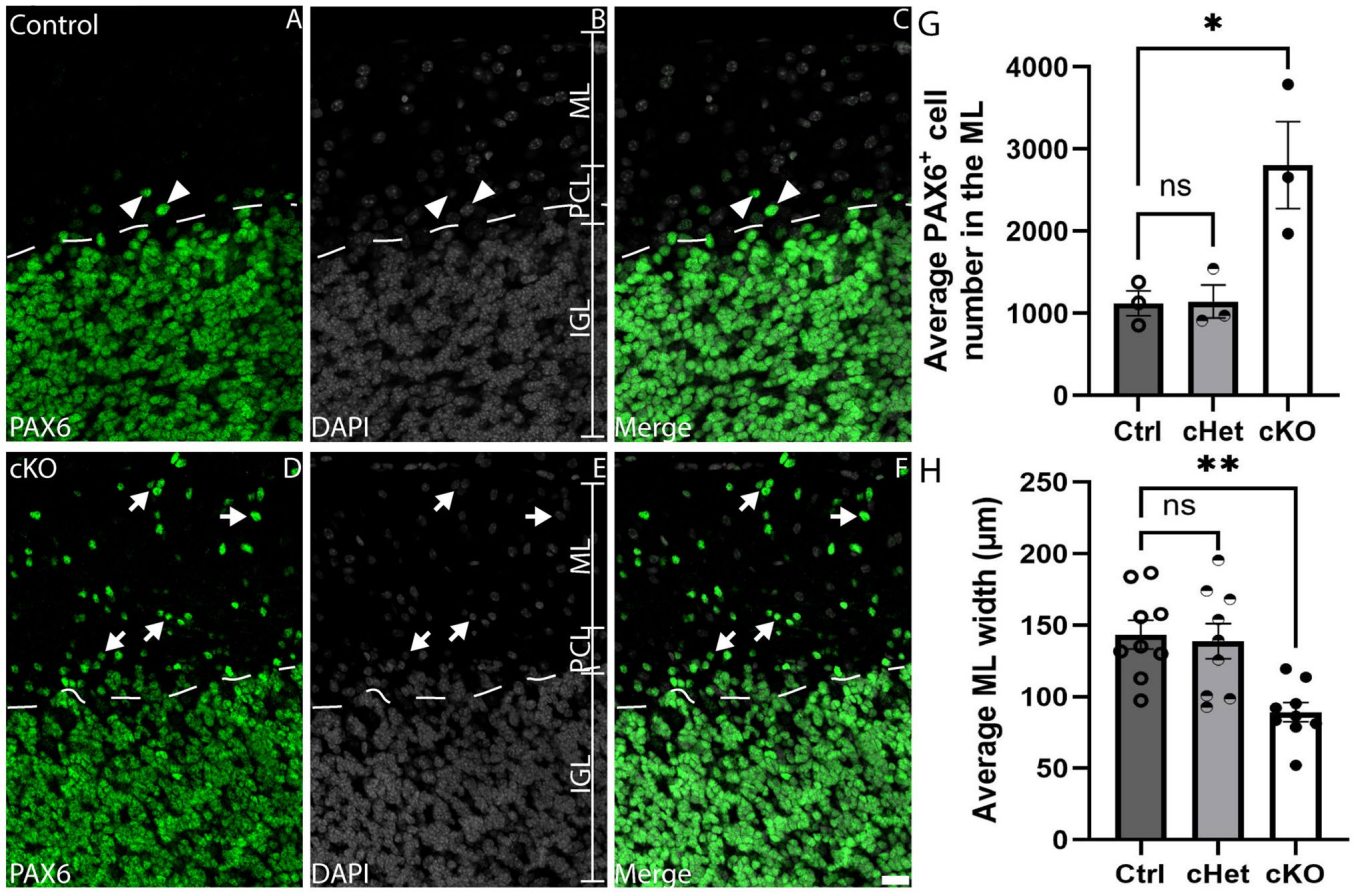
Abnormal migration of developing granule neurons in the absence of *Tead1*. Mid-sagittal sections of control (**A-C**) and cKO (**D-F**) cerebella at P20, revealing the expression of PAX6 (green); nuclei were labelled with DAPI (grey). In control cerebella, PAX6 expression was predominantly confined to the IGL, with relatively few PAX6-expressing cells evident within the ML (arrowheads in **A**-**C**). The dotted lines demarcate the boundary between the PCL and the IGL. In comparison, many more PAX6-expressing cells were evident within the ML of cKO cerebella (arrows in **D**-**F**, quantified in **G**). Furthermore, the width of the ML was significantly reduced in cKO mice (**H**). Scale bar (in F) represents 20 µm for all panels. ns, not significant, * *p* < 0.05, ** *p* < 0.01; one-way ANOVA

BG fibres are also critical for the migration of newborn granule neurons to the IGL. We used the expression of PAX6 to identify granule neurons within the cerebellum of P20 mice to determine if their migration was disrupted in cKO mice. At P20, PAX6 expression was primarily confined to the IGL in control mice, suggestive of granule neurons having completed their migration (Fig. 10A-C). By comparison, there were significantly more PAX6-expressing cells within the ML of cKO mice, suggestive of delayed migration of newborn granule neuron cells into the IGL (Fig. 10D-G). This phenotype was seen despite the significantly reduced width of the ML evident in cKO mice (Fig. 10H), a phenotype potentially arising from reduced PN dendritogenesis or aberrant BG development (Cerrato et al. 2018; Cheng et al. 2018; van der Heijden and Sillitoe 2021). Taken altogether, these findings suggest that granule neurons exhibit abnormal migration in the absence of *Tead1*, indicative of another non-cell-autonomous defect in these cKO mice.

## Discussion

Transcriptional regulation of gene expression is crucial for organogenesis (Mukhtar et al. 2022; Xue et al. 2011; Papadimitriou and Thomaidou 2024). Previous studies have begun to elucidate how Hippo signalling, an evolutionarily conserved organogenesis pathway (Chen et al. 2020) functions in a number of different organ systems, such as the liver (Russell and Camargo 2022), heart (Mia and Singh 2019) and lungs (Lange et al. 2015). However, the role of Hippo in nervous system development remains unclear. While some studies have highlighted the role of YAP/TAZ in nervous system development (Hughes et al. 2020; Park et al. 2016), our understanding of the role of the YAP/TAZ-interacting transcription factors, the TEADs is limited. Further, investigations have primarily focussed on the role of Hippo-TEAD in corticogenesis (Mukhtar et al. 2020), with research into the cerebellum limited. Here, we use the postnatal developing cerebellum as a model to probe the spatiotemporal expression of TEAD1, but also how cerebellar development is dysregulated in the absence of this transcription co-factor. We reveal that TEAD1 is expressed by CGNPs in the early postnatal cerebellum, as well as by glial progenitor cells and astrocytes/BG. We also reveal both cell- and non-cell-autonomous roles for TEAD1 in formation of the cerebellum.

Recently, YAP/TAZ function was shown to be important for cerebellar development (Hughes et al. 2020). The authors revealed expression of these Hippo components within glial cells and CGNPs, but not within PNs. Our data are consistent with these findings, suggesting that TEAD1 is a key component of Hippo/YAP/TAZ signalling within the emerging cerebellum. Moreover, our data mirror that of Hughes and colleagues, who used a double *Yap/Taz* conditional ablation approach to disrupt Hippo signalling. Their data revealed foliation deficits and reduced cerebellar size, akin to our findings. *Yap/Taz* mutants also exhibited abnormal BG development, suggestive of cell-autonomous BG deficits, like our cKO mice. These data suggest that YAP/TAZ signalling within the developing cerebellum is regulated, at least in part, by TEAD1 function.

One of the key findings of our study was that glial progenitor cells and BG express TEAD1, and that the development of BG fibres is abnormal in cKO mice. This is suggestive of a cell-autonomous role for TEAD1 within these cells. What role could Hippo/TEAD1 signalling play in BG development? One possibility is that Hippo signalling could mediate PTEN phosphatase activity. This factor plays a crucial role in the intrinsic specification and regulation of BG differentiation and scaffold organisation (Yue et al. 2005). Importantly, miR-29 has been shown to regulate PTEN expression (Tumaneng et al. 2012), and, moreover, TEAD1 has been shown to drive *Yap/Taz-*dependent transcription of the micro-RNA miR-29a/b/c family (Tumaneng et al. 2012). While these studies were not performed in the context of the developing cerebellum, they do suggest one potential mechanism by which TEAD1 could promote BG development. BG also play crucial roles in foliation, not only through regulation of granule neuron migration (Consalez et al. 2020), but also through PN dendritogenesis and synaptogenesis (Bellamy 2006; Lippman et al. 2008; Ango et al. 2008). As such, dysregulation of BG development may help to explain the deficits seen in our cKO mice, such as delayed granule neuron migration and PN dendritogenesis. Looking ahead, single cell sequencing of the cerebella of cKO mice could reveal transcriptional deficits in different cellular populations, such as BG. Furthermore, as the binding motif of the TEAD family is known (Filandrova et al. 2021), potential direct TEAD1 targets could be identified, and their role downstream of TEAD1 assessed using genetic approaches, such as the analysis of trans-heterozygotes, or the manipulation of TEAD1 target gene expression specifically within BG.

Our data also reveal TEAD1 expression within CGNPs, suggestive of a potential role for Hippo signalling in mediating proliferation within this germinal niche. However, deletion of both *Yap* and *Taz* in CGNPs has previously been investigated, revealing no proliferative deficits in postnatal CGNPs, either in vitro or in vivo, even when these factors were ablated specifically from CGNP’s using a *Math1-Cre*-based conditional approach (Hughes et al. 2020). Our data mirrored these findings; we did not observe any significant changes in the number (Welch’s *t*-test, *p* = 0.6666) or density (Welch’s *t*-test, *p* = 0.7983) of proliferative cells within the EGL of the cKO in comparison to the control at P5. What then is the role for Hippo pathway activity in these cells? It has been recently demonstrated that TEAD1 has roles independent for proliferation, meaning that binding of YAP/TAZ is not required for it to exert proliferative effects in all contexts (Li et al. 2022). One potential role for TEAD1 activity on these cells could be to mediate the development of PNs. CGNPs are known to regulate PN dendrite development via WNT signalling; aberrant CGNP-derived WNT signalling can lead to both disrupted PN dendritic development as well as altered PN soma localisation (Cheng et al. 2018). In future, investigation of altered signalling from *Tead1-*deficient CGNPs, potentially via transcriptomic-based experiments, could help define the cell-autonomous role of this transcription factor within these cells. Analysis of PN dendrite morphology and synapse formation, perhaps via Golgi-Cox analysis, could also help elucidate how TEAD1 impacts this cellular population in a non-cell-autonomous manner.

One limitation of our study was that *Tead1* was ablated from neural stem cells throughout the neuraxis from E8, making it difficult to parse effects we see within the postnatal cerebellum from earlier developmental events. One way to mitigate this in future could be to use different Cre lines, enabling removal of *Tead1* specifically from different embryonic and postnatal cellular populations. An example of this could be a *Sox2-CreER*^*T2*^ line, as SOX2 is expressed by glial cells including BG. Postnatal tamoxifen administration could enable the role of TEAD1 within the cerebellum at this age to be differentiated from any embryonic roles. Moreover, this approach could provide a mechanism to test both the cell-autonomous roles within BG, and non-cell-autonomous roles of TEAD1 within the cerebellum, as has been similarly done previously (Cerrato et al. 2018). If TEAD1 expression within BG contributes to the non-cell-autonomous development of PNs, we would posit that ablating *Tead1* from BG would phenocopy the PN deficits we observed in our broader conditional approach. Single cell sequencing of BG within such a *Tead1; Sox2-CreER*^*T2*^ line could also identify the mechanistic processes underpinning both BG development, and the cell–cell signalling processes controlled by Hippo signalling that regulate PN morphogenesis. Rescue experiments in this line could further clarify the molecular mechanisms whereby TEAD1-dependent BG development controls PN formation. Similarly, the use of a *Math1-CreER*^*T2*^ line (Machold and Fishell 2005) would enable the role of TEAD1 within CGNPs, both embryonically and postnatally, to be analysed, defining both the cell-autonomous, and non-cell-autonomous roles of this factor within these cells. Collectively, our study reveals a range of cell- and non-cell-autonomous roles for TEAD1 within the postnatal cerebellum, highlighting the critical role for Hippo signalling in the formation of this critical brain region.

## Supplementary Information

Below is the link to the electronic supplementary material.

Supplementary file1 (DOCX 30 kb)

Supplementary file2 (TIF 17776 kb)

Supplementary file3 (TIF 12118 kb)

Supplementary file4 (TIF 28976 kb)

Supplementary file5 (TIF 9927 kb)

Supplementary file6 (TIF 21127 kb)

## Data Availability

No datasets were generated or analysed during the current study.

## References

[CR1] Amore G, Spoto G, Ieni A, Vetri L, Quatrosi G, Di Rosa G et al (2021) A focus on the cerebellum: from embryogenesis to an age-related clinical perspective. Front Syst Neurosci 15:646052 33897383 10.3389/fnsys.2021.646052 PMC8062874

[CR2] Ango F, Wu C, Van der Want JJ, Wu P, Schachner M, Huang ZJ (2008) Bergmann glia and the recognition molecule CHL1 organize GABAergic axons and direct innervation of Purkinje cell dendrites. PLoS Biol 6(4):e103 18447583 10.1371/journal.pbio.0060103 PMC2689695

[CR3] Araujo APB, Carpi-Santos R, Gomes FCA (2019) The role of astrocytes in the development of the cerebellum. Cerebellum 18(6):1017–1035 31218566 10.1007/s12311-019-01046-0

[CR4] Bastianelli E (2003) Distribution of calcium-binding proteins in the cerebellum. Cerebellum 2(4):242–262 14964684 10.1080/14734220310022289

[CR5] Bellamy TC (2006) Interactions between Purkinje neurones and Bergmann glia. Cerebellum 5(2):116–126 16818386 10.1080/14734220600724569

[CR6] Bhattacharya D, Krummel DP, Sengupta S (2020) Pediatric cerebellar tumors: transcriptionally distinct but developmentally heterogeneous. Transl Cancer Res 9(3):1322–1325 35117480 10.21037/tcr.2020.01.31 PMC8798252

[CR7] Brawand D, Soumillon M, Necsulea A, Julien P, Csardi G, Harrigan P et al (2011) The evolution of gene expression levels in mammalian organs. Nature 478(7369):343–348 22012392 10.1038/nature10532

[CR8] Bui DA, Lee W, White AE, Harper JW, Schackmann RC, Overholtzer M et al (2016) Cytokinesis involves a nontranscriptional function of the Hippo pathway effector YAP. Sci Signal 9(417):23 10.1126/scisignal.aaa9227 PMC5455055 26933062

[CR9] Cebola I, Rodriguez-Segui SA, Cho CH, Bessa J, Rovira M, Luengo M et al (2015) TEAD and YAP regulate the enhancer network of human embryonic pancreatic progenitors. Nat Cell Biol 17(5):615–626 25915126 10.1038/ncb3160 PMC4434585

[CR10] Cerrato V, Mercurio S, Leto K, Fuca E, Hoxha E, Bottes S et al (2018) Sox2 conditional mutation in mouse causes ataxic symptoms, cerebellar vermis hypoplasia, and postnatal defects of Bergmann glia. Glia 66(9):1929–1946 29732603 10.1002/glia.23448

[CR11] Chen Y, Han H, Seo G, Vargas RE, Yang B, Chuc K et al (2020) Systematic analysis of the Hippo pathway organization and oncogenic alteration in evolution. Sci Rep 10(1):3173 32081887 10.1038/s41598-020-60120-4 PMC7035326

[CR12] Cheng FY, Fleming JT, Chiang C (2018) Bergmann glial Sonic hedgehog signaling activity is required for proper cerebellar cortical expansion and architecture. Dev Biol 440(2):152–166 29792854 10.1016/j.ydbio.2018.05.015 PMC6014626

[CR13] Chizhikov V, Millen KJ (2003) Development and malformations of the cerebellum in mice. Mol Genet Metab 80(1–2):54–65 14567957 10.1016/j.ymgme.2003.08.019

[CR14] International Mouse Knockout C, Collins FS, Rossant J, Wurst W. A mouse for all reasons. Cell. 2007;128(1):9–13. 10.1016/j.cell.2006.12.018 17218247

[CR15] Consalez GG, Hawkes R (2012) The compartmental restriction of cerebellar interneurons. Front Neural Circuits 6:123 23346049 10.3389/fncir.2012.00123 PMC3551280

[CR16] Consalez GG, Goldowitz D, Casoni F, Hawkes R (2020) Origins, development, and compartmentation of the granule cells of the cerebellum. Front Neural Circuits 14:611841 33519389 10.3389/fncir.2020.611841 PMC7843939

[CR17] Currey L, Thor S, Piper M (2021) TEAD family transcription factors in development and disease. Development 148(12):196675 10.1242/dev.196675 34128986

[CR18] D’Arrigo S, Loiacano C, Ciaccio C, Pantaleoni C, Faccio F, Taddei M, Bulgheroni S (2021) Clinical, cognitive and behavioural assessment in children with cerebellar disorder. Appl Sci 11(2):544

[CR19] De Luca A, Parmigiani E, Tosatto G, Martire S, Hoshino M, Buffo A et al (2015) Exogenous Sonic hedgehog modulates the pool of GABAergic interneurons during cerebellar development. Cerebellum 14(2):72–85 25245619 10.1007/s12311-014-0596-x

[CR20] Englund C, Kowalczyk T, Daza RA, Dagan A, Lau C, Rose MF et al (2006) Unipolar brush cells of the cerebellum are produced in the rhombic lip and migrate through developing white matter. J Neurosci 26(36):9184–9195 16957075 10.1523/JNEUROSCI.1610-06.2006 PMC6674506

[CR21] Feng X, Wang Z, Wang F, Lu T, Xu J, Ma X et al (2019) Dual function of VGLL4 in muscle regeneration. EMBO J 38(17):e101051 31328806 10.15252/embj.2018101051 PMC6717915

[CR22] Filandrova R, Valis K, Cerny J, Chmelik J, Slavata L, Fiala J et al (2021) Motif orientation matters: Structural characterization of TEAD1 recognition of genomic DNA. Structure 29(4):345–56.e8 33333006 10.1016/j.str.2020.11.018

[CR23] Fraser J, Essebier A, Gronostajski RM, Boden M, Wainwright BJ, Harvey TJ et al (2017) Cell-type-specific expression of NFIX in the developing and adult cerebellum. Brain Struct Funct 222(5):2251–2270 27878595 10.1007/s00429-016-1340-8

[CR24] Gee ST, Milgram SL, Kramer KL, Conlon FL, Moody SA (2011) Yes-associated protein 65 (YAP) expands neural progenitors and regulates Pax3 expression in the neural plate border zone. PLoS ONE 6(6):e20309 21687713 10.1371/journal.pone.0020309 PMC3110623

[CR25] Haldipur P, Dang D, Millen KJ (2018) Embryology. Handb Clin Neurol 154:29–44 29903446 10.1016/B978-0-444-63956-1.00002-3 PMC6231496

[CR26] Honda M, Tsuchimochi H, Hitachi K, Ohno S (2019) Transcriptional cofactor Vgll2 is required for functional adaptations of skeletal muscle induced by chronic overload. J Cell Physiol 234(9):15809–15824 30724341 10.1002/jcp.28239

[CR27] Huff J (2015) The Airyscan detector from ZEISS: confocal imaging with improved signal-to-noise ratio and super-resolution. Nat Methods 12(12):i–ii

[CR28] Huff J. The Airyscan Detector: Confocal Microscopy Evolution for the Neurosciences. 2019. In: Advanced Optical Methods for Brain Imaging [Internet]. Progress in Optical Science and Photonics: Springer, Singapore; [83–102].

[CR29] Hughes LJ, Park R, Lee MJ, Terry BK, Lee DJ, Kim H et al (2020) Yap/Taz are required for establishing the cerebellar radial glia scaffold and proper foliation. Dev Biol 457(1):150–162 31586559 10.1016/j.ydbio.2019.10.002 PMC6910221

[CR30] Iskusnykh IY, Chizhikov VV (2022) Cerebellar development after preterm birth. Front Cell Dev Biol 10:1068288 36523506 10.3389/fcell.2022.1068288 PMC9744950

[CR31] Kalinichenko SG, Okhotin VE (2005) Unipolar brush cells–a new type of excitatory interneuron in the cerebellar cortex and cochlear nuclei of the brainstem. Neurosci Behav Physiol 35(1):21–36 15739785 10.1023/b:neab.0000049648.20702.ad

[CR32] Kaneko KJ, Kohn MJ, Liu C, DePamphilis ML (2007) Transcription factor TEAD2 is involved in neural tube closure. Genesis 45(9):577–587 17868131 10.1002/dvg.20330 PMC2765819

[CR33] Kimura M, Horie T, Baba O, Ide Y, Tsuji S, Ruiz Rodriguez R et al (2020) Homeobox A4 suppresses vascular remodeling by repressing YAP/TEAD transcriptional activity. EMBO Rep 21(4):e48389 32147946 10.15252/embr.201948389 PMC7132199

[CR34] Kwon H, Kim J, Jho EH (2022) Role of the Hippo pathway and mechanisms for controlling cellular localization of YAP/TAZ. FEBS J 289(19):5798–5818 34173335 10.1111/febs.16091

[CR35] Lange AW, Sridharan A, Xu Y, Stripp BR, Perl AK, Whitsett JA (2015) Hippo/Yap signaling controls epithelial progenitor cell proliferation and differentiation in the embryonic and adult lung. J Mol Cell Biol 7(1):35–47 25480985 10.1093/jmcb/mju046 PMC4400400

[CR36] Lee HY, Greene LA, Mason CA, Manzini MC. Isolation and culture of post-natal mouse cerebellar granule neuron progenitor cells and neurons. J Vis Exp. 2009(23). 10.3791/990 PMC2781826 19229177

[CR37] Leto K, Bartolini A, Rossi F (2008) Development of cerebellar GABAergic interneurons: origin and shaping of the “minibrain” local connections. Cerebellum 7(4):523–529 19002744 10.1007/s12311-008-0079-z

[CR38] Leto K, Rolando C, Rossi F (2012) The genesis of cerebellar GABAergic neurons: fate potential and specification mechanisms. Front Neuroanat 6:6 22363268 10.3389/fnana.2012.00006 PMC3282257

[CR39] Leto K, Arancillo M, Becker EB, Buffo A, Chiang C, Ding B et al (2016) Consensus paper: cerebellar development. Cerebellum 15(6):789–828 26439486 10.1007/s12311-015-0724-2 PMC4846577

[CR40] Li F, Negi V, Yang P, Lee J, Ma K, Moulik M et al (2022) TEAD1 regulates cell proliferation through a pocket-independent transcription repression mechanism. Nucleic Acids Res 50(22):12723–12738 36484096 10.1093/nar/gkac1063 PMC9825168

[CR41] Lippman JJ, Lordkipanidze T, Buell ME, Yoon SO, Dunaevsky A (2008) Morphogenesis and regulation of Bergmann glial processes during Purkinje cell dendritic spine ensheathment and synaptogenesis. Glia 56(13):1463–1477 18615636 10.1002/glia.20712 PMC2637407

[CR42] Machold R, Fishell G (2005) Math1 is expressed in temporally discrete pools of cerebellar rhombic-lip neural progenitors. Neuron 48(1):17–24 16202705 10.1016/j.neuron.2005.08.028

[CR43] Machold R, Klein C, Fishell G (2011) Genes expressed in Atoh1 neuronal lineages arising from the r1/isthmus rhombic lip. Gene Expr Patterns 11(5–6):349–359 21440680 10.1016/j.gep.2011.03.007 PMC3095718

[CR44] Malik AR, Liszewska E, Jaworski J (2015) Matricellular proteins of the Cyr61/CTGF/NOV (CCN) family and the nervous system. Front Cell Neurosci 9:237 26157362 10.3389/fncel.2015.00237 PMC4478388

[CR45] Manto M, Bower JM, Conforto AB, Delgado-Garcia JM, da Guarda SN, Gerwig M et al (2012) Consensus paper: roles of the cerebellum in motor control–the diversity of ideas on cerebellar involvement in movement. Cerebellum 11(2):457–487 22161499 10.1007/s12311-011-0331-9 PMC4347949

[CR46] Weigert MaS, Uwe. Nuclei instance segmentation and classification in histopathology images with StarDist. The IEEE International Symposium on Biomedical Imaging Challenges (ISBIC); Kolkata, India, 2022.

[CR47] Mia MM, Singh MK (2019) The Hippo signaling pathway in cardiac development and diseases. Front Cell Dev Biol 7:211 31632964 10.3389/fcell.2019.00211 PMC6779857

[CR48] Misra JR, Irvine KD (2018) The Hippo signaling network and its biological functions. Annu Rev Genet 52:65–87 30183404 10.1146/annurev-genet-120417-031621 PMC6322405

[CR49] Mukhtar T, Breda J, Grison A, Karimaddini Z, Grobecker P, Iber D et al (2020) Tead transcription factors differentially regulate cortical development. Sci Rep 10(1):4625 32170161 10.1038/s41598-020-61490-5 PMC7070074

[CR50] Mukhtar T, Breda J, Adam MA, Boareto M, Grobecker P, Karimaddini Z et al (2022) Temporal and sequential transcriptional dynamics define lineage shifts in corticogenesis. EMBO J 41(24):e111132 36345783 10.15252/embj.2022111132 PMC9753470

[CR51] Oishi S, Premaranthne, S, Harvey, TJ, Iyer, S, Dixon, C, Alexander, S, Burne, THJ, Wood, SA & Piper, M. Usp9x-deficiency disrupts the morphological development of the postnatal hippocampal dentate gyrus. Scientific Reports. 2016;6. 10.1038/srep25783 PMC4867638 27181636

[CR52] Osman I, He X, Liu J, Dong K, Wen T, Zhang F et al (2019) TEAD1 (TEA Domain Transcription Factor 1) Promotes Smooth Muscle Cell Proliferation Through Upregulating SLC1A5 (Solute Carrier Family 1 Member 5)-Mediated Glutamine Uptake. Circ Res 124(9):1309–1322 30801233 10.1161/CIRCRESAHA.118.314187 PMC6493685

[CR53] Packer RJ, Cogen P, Vezina G, Rorke LB (1999) Medulloblastoma: clinical and biologic aspects. Neuro Oncol 1(3):232–250 11550316 10.1215/15228517-1-3-232 PMC1920747

[CR54] Papadimitriou E, Thomaidou D (2024) Post-transcriptional mechanisms controlling neurogenesis and direct neuronal reprogramming. Neural Regen Res 19(9):1929–1939 38227517 10.4103/1673-5374.390976 PMC11040297

[CR55] Park R, Moon UY, Park JY, Hughes LJ, Johnson RL, Cho SH et al (2016) Yap is required for ependymal integrity and is suppressed in LPA-induced hydrocephalus. Nat Commun 7:10329 26754915 10.1038/ncomms10329 PMC4729961

[CR56] Pelenyi A, Atterton C, Jones J, Currey L, Al-Khalily M, Wright L et al (2024) Expression of the Hippo pathway effector, TEAD1, within the developing murine forebrain. Gene Expr Patterns 54:119384 39557142 10.1016/j.gep.2024.119384

[CR57] Piper M, Barry G, Hawkins J, Mason S, Lindwall C, Little E et al (2010) NFIA controls telencephalic progenitor cell differentiation through repression of the Notch effector Hes1. J Neurosci 30(27):9127–9139 20610746 10.1523/JNEUROSCI.6167-09.2010 PMC6632468

[CR58] Piper M, Harris L, Barry G, Heng YH, Plachez C, Gronostajski RM et al (2011) Nuclear factor one X regulates the development of multiple cellular populations in the postnatal cerebellum. J Comp Neurol 519(17):3532–3548 21800304 10.1002/cne.22721 PMC4032092

[CR59] Rausch V, Hansen CG (2020) The Hippo pathway, YAP/TAZ, and the plasma membrane. Trends Cell Biol 30(1):32–48 31806419 10.1016/j.tcb.2019.10.005

[CR60] Ribas R, Moncaut N, Siligan C, Taylor K, Cross JW, Rigby PW et al (2011) Members of the TEAD family of transcription factors regulate the expression of Myf5 in ventral somitic compartments. Dev Biol 355(2):372–380 21527258 10.1016/j.ydbio.2011.04.005 PMC3123743

[CR61] Rueden CT, Schindelin J, Hiner MC, DeZonia BE, Walter AE, Arena ET et al (2017) Image J2: ImageJ for the next generation of scientific image data. BMC Bioinformatics 18(1):529 29187165 10.1186/s12859-017-1934-z PMC5708080

[CR62] Russell JO, Camargo FD (2022) Hippo signalling in the liver: role in development, regeneration and disease. Nat Rev Gastroenterol Hepatol 19(5):297–312 35064256 10.1038/s41575-021-00571-w PMC9199961

[CR63] Sahu MR, Mondal AC (2021) Neuronal Hippo signaling: From development to diseases. Dev Neurobiol 81(2):92–109 33275833 10.1002/dneu.22796

[CR64] Schmidt U, Weigert, Martin., Broaddus, Coleman and Myers, Gene. Cell Detection with Star-convex Polygons. International Conference on Medical Image Computing and Computer-Assisted Intervention (MICCAI); Granada, Spain. 2018.

[CR65] Shreberk-Shaked M, Oren M (2019) New insights into YAP/TAZ nucleo-cytoplasmic shuttling: new cancer therapeutic opportunities? Mol Oncol 13(6):1335–1341 31050214 10.1002/1878-0261.12498 PMC6547617

[CR66] Skarnes WC, Rosen B, West AP, Koutsourakis M, Bushell W, Iyer V et al (2011) A conditional knockout resource for the genome-wide study of mouse gene function. Nature 474(7351):337–342 21677750 10.1038/nature10163 PMC3572410

[CR67] Stoodley CJ (2016) The cerebellum and neurodevelopmental disorders. Cerebellum 15(1):34–37 26298473 10.1007/s12311-015-0715-3 PMC4811332

[CR68] Sun C, De Mello V, Mohamed A, Ortuste Quiroga HP, Garcia-Munoz A, Al Bloshi A et al (2017) Common and distinctive functions of the hippo effectors taz and yap in skeletal muscle stem cell function. Stem Cells 35(8):1958–1972 28589555 10.1002/stem.2652 PMC5575518

[CR69] Takashima Y, Era T, Nakao K, Kondo S, Kasuga M, Smith AG et al (2007) Neuroepithelial cells supply an initial transient wave of MSC differentiation. Cell 129(7):1377–1388 17604725 10.1016/j.cell.2007.04.028

[CR70] Terry BK, Kim S (2022) The role of Hippo-YAP/TAZ signaling in brain development. Dev Dyn 251(10):1644–1665 35651313 10.1002/dvdy.504

[CR71] Tumaneng K, Schlegelmilch K, Russell RC, Yimlamai D, Basnet H, Mahadevan N et al (2012) YAP mediates crosstalk between the Hippo and PI(3)K-TOR pathways by suppressing PTEN via miR-29. Nat Cell Biol 14(12):1322–1329 23143395 10.1038/ncb2615 PMC4019071

[CR72] Ullmann JF, Keller MD, Watson C, Janke AL, Kurniawan ND, Yang Z et al (2012) Segmentation of the C57BL/6J mouse cerebellum in magnetic resonance images. Neuroimage 62(3):1408–1414 22658976 10.1016/j.neuroimage.2012.05.061

[CR73] van der Heijden ME, Sillitoe RV (2021) Interactions between purkinje cells and granule cells coordinate the development of functional cerebellar circuits. Neuroscience 462:4–21 32554107 10.1016/j.neuroscience.2020.06.010 PMC7736359

[CR74] van der Heijden ME, Gill JS, Sillitoe RV (2021) Abnormal cerebellar development in autism spectrum disorders. Dev Neurosci 43(3–4):181–190 33823515 10.1159/000515189 PMC8440334

[CR75] Wang L, Liu Y (2019) Signaling pathways in cerebellar granule cells development. Am J Stem Cells 8(1):1–6 31139492 PMC6526362

[CR76] Wang W, Shiraishi R, Kawauchi D (2022) Sonic hedgehog signaling in cerebellar development and cancer. Front Cell Dev Biol 10:864035 35573667 10.3389/fcell.2022.864035 PMC9100414

[CR77] Weigert M, Schmidt, Uwe., Haase, Robert., Sugawara, Ko and Myers, Gene. Star-convex Polyhedra for 3D Object Detection and Segmentation in Microscopy. The IEEE Winter Conference on Applications of Computer Vision (WACV); Snowmass Village, Colorado. 2020.

[CR78] Wen T, Liu J, He X, Dong K, Hu G, Yu L et al (2019) Transcription factor TEAD1 is essential for vascular development by promoting vascular smooth muscle differentiation. Cell Death Differ 26(12):2790–2806 31024075 10.1038/s41418-019-0335-4 PMC7224394

[CR79] Wierzba-Bobrowicz T, Lewandowska E, Stepien T, Szpak GM (2011) Differential expression of calbindin D28k, calretinin and parvalbumin in the cerebellum of pups of ethanol-treated female rats. Folia Neuropathol 49(1):47–55 21455843

[CR80] Xue L, Yi H, Huang Z, Shi YB, Li WX (2011) Global gene expression during the human organogenesis: from transcription profiles to function predictions. Int J Biol Sci 7(7):1068–1076 21927576 10.7150/ijbs.7.1068 PMC3174391

[CR81] Yamada K, Watanabe M (2002) Cytodifferentiation of Bergmann glia and its relationship with Purkinje cells. Anat Sci Int 77(2):94–108 12418089 10.1046/j.0022-7722.2002.00021.x

[CR82] Yue Q, Groszer M, Gil JS, Berk AJ, Messing A, Wu H et al (2005) PTEN deletion in Bergmann glia leads to premature differentiation and affects laminar organization. Development 132(14):3281–3291 15944184 10.1242/dev.01891

