# Supplementary material for: The Hippo effector TEAD1 regulates postnatal murine cerebellar development
Source: Brain Struct Funct. 2025 Mar 10;230(3):42. doi: 10.1007/s00429-025-02903-x (PMC11893647; doi:10.1007/s00429-025-02903-x)
Supplement: Supplementary file 1 — Supplementary file1 (DOCX 30 kb) [file 429_2025_2903_MOESM1_ESM.docx]

**The Hippo effector TEAD1 regulates postnatal murine cerebellar development**

Cooper Atterton^1^, Alexandra Pelenyi^1^, Justin Jones^1^, Laura Currey^1^, Majd Al-Khalily^2^, Lucinda Wright^1^, Mikki Doonan^1^, David Knight^1^, Nyoman D. Kurniawan^2^, Shaun Walters^1^, Stefan Thor^1^ and Michael Piper^1, 3, *^

^1^ The School of Biomedical Sciences, Faculty of Medicine, The University of Queensland, QLD, 4072, Australia.

^2^ The Centre for Advanced Imaging, The University of Queensland, QLD, 4072, Australia.

^3^ The Queensland Brain Institute, The University of Queensland, QLD, 4072, Australia.

^*^ Corresponding author:

Michael Piper, The School of Biomedical Sciences, The University of Queensland, QLD, 4072, Australia

Email address: m.piper@uq.edu.au

Keywords: TEAD1, Hippo, cerebellum, granule cell, Purkinje neuron, Bergmann glia

**Acknowledgements**

The work was funded by grants from the Australian Research Council (DP220100985 and DP230101750) to MP and ST, and a grant from the National Health and Medical Research Council of Australia to ST. CA and AP were supported by Research Training Program stipends from the Australian Government. We acknowledge the supports from the Queensland NMR Network and the National Imaging Facility (a National Collaborative Research Infrastructure Strategy capability) for the operation of 16.4 T MRI at the Centre for Advanced Imaging. Finally, we thank the animal team from UQ Biological Resources (QBI) for their exceptional care and housing of our animals.

**SUPPLEMENTARY DATA**

**Supplementary Table 1:** **Software packages used for analyses.** List of software packages used for volumetric and statistical analyses.

| **Software Package Name** | **Software Package Source** | **Software Package Purpose** |
| --- | --- | --- |
| FSL | https://fsl.fmrib.ox.ac.uk/fsl/fslwiki | Linear atlas registration to cerebellum |
| ANTS | https://stnava.github.io/ANTs/ | Warped/deformation atlas registration to cerebellum |
| ITKSnap | http://www.itksnap.org/pmwiki/pmwiki.php | Segment structures and adjust templates ROIs to match anatomical regions |
| GraphPad Prism | N/A | Statistical analyses |

**Supplementary Table 2: Full volumetric analyses**. Results from full volumetric analyses of whole cerebellum volume, as well as individual regions as defined by the applied template. Volumes are represented as mm^3^, and the difference in volume between control and cHet samples is shown (a positive volume means the control is larger, whereas a negative value means the cHet is larger). Students t-test with Holm-Sidak correction was performed to determine significance of volumetric differences between regions in control and cHet mice and is represented as an adjusted p-value. Green p-values are significantly different (p < 0.05), while red p-values did not reach significance (p > 0.05). Abbreviations: Whole cerebellum – **WC**; Internal capsule – **IC**; Lobule 1 – **1Cb**; Lobule 1/2 – **1/2Cb**; Lobule 2 – **2Cb**; Lobule 2/3 – **2/3Cb**; Lobule 3 – **3Cb**; Lobule 3/4 – **3/4Cb**; Lobule 4 – **4Cb**; Lobule 4/5 – **4/5Cb**; Lobule 5 – **5Cb**; Lobule 6 – **6Cb**; Lobule 7 – **7Cb**; Lobule 8 – **8Cb**; Lobule 9 – **9Cb**; Lobule 10 – **10Cb**; Simple lobule – **Sim**; Crus 1 of the ansiform lobule – **Crus1**; Crus 2 of the ansiform lobule – **Crus2**; Paramedian lobule – **PM**; Copula of the pyramis – **Cop**; Paraflocculus – **PFl**; Flocculus – **Fl**; Medial longitudinal fasciculus – **mlf**; Superior cerebellar peduncle – **scp**; Middle cerebellar peduncle – **mcp**; Inferior cerebellar peduncle – **icp**; Decussation of the superior cerebellar peduncle – **xscp**; Medial forebrain bundle – **mfb**; Rhabdoid nucleus – **Rbd**; Vermal lingula anterior – **VLA**; Ventral spinocerebellar tract – **vsc**; Medial cerebellar nucleus – **Med**; Medial cerebellar nucleus, dorsolateral protuberance – **MedDL**; Medial cerebellar nucleus, lateral part – **MedL**; Lateral cerebellar nucleus – **Lat**; Lateral cerebellar nucleus, parvicellular part – **LatPC**; Superior medullary velum – **SMV**; Dorsal cochlear nuclei – **DC**; Ventral cochlear nuclei, anterior part – **VCA**; Ventral cochlear nuclei, posterior part – **VCP**; Interposed cerebellar nucleus, anterior part – **IntA**; Interposed cerebellar nucleus, dorsolateral hump – **IntDL**; Interposed cerebellar nucleus, posterior part – **IntP**; Interposed cerebellar nucleus, posterior parvicellular part – **IntPPC**; Dorsal acoustic stria – **das**.

| Structure Volume (mm^3^) | | | | |
| --- | --- | --- | --- | --- |
| *Region* | **Mean of Tead1 Ctrls** | **Mean of Tead1 cHets** | **Difference in Volume (Ctrl – cHet)** | **Adjusted p-value** |
| *WC* | 54.460 | 48.015 | 6.445 | 0.002 |
| *IC* | 0.004370 | 0.004741 | -0.000371 | 0.907511 |
| *1Cb* | 0.2741 | 0.2406 | 0.0335 | 0.155803 |
| *1/2Cb* | 0.2279 | 0.1916 | 0.0363 | 0.296114 |
| *2Cb* | 1.228 | 1.074 | 0.154 | 0.396825 |
| *2/3Cb* | 0.4613 | 0.4073 | 0.054 | 0.127541 |
| *3Cb* | 1.690 | 1.450 | 0.24 | 0.162726 |
| *3/4Cb* | 0.07707 | 0.07093 | 0.00614 | 0.582147 |
| *4Cb* | 0.02415 | 0.02007 | 0.00408 | 0.066409 |
| *4/5Cb* | 5.462 | 4.813 | 0.649 | 0.037702 |
| *5Cb* | 0.2120 | 0.1940 | 0.018 | 0.137737 |
| *6Cb* | 3.001 | 2.638 | 0.363 | 0.039797 |
| *7Cb* | 0.6999 | 0.6165 | 0.0834 | 0.398435 |
| *8Cb* | 1.489 | 1.209 | 0.28 | 0.162087 |
| *9Cb* | 2.553 | 1.962 | 0.591 | 0.092416 |
| *10Cb* | 1.076 | 0.8824 | 0.1936 | 0.162087 |
| *Sim* | 4.810 | 4.239 | 0.571 | 0.117224 |
| *Crus1* | 4.886 | 4.228 | 0.658 | 0.083342 |
| *Crus2* | 4.953 | 4.218 | 0.735 | 0.037702 |
| *PM* | 4.107 | 3.625 | 0.482 | 0.137737 |
| *Cop* | 2.363 | 2.000 | 0.363 | 0.044369 |
| *PFI* | 4.420 | 3.989 | 0.431 | 0.582147 |
| *FL* | 0.8326 | 0.7758 | 0.0568 | 0.582147 |
| *mlf* | 0.09678 | 0.08459 | 0.01219 | 0.296711 |
| *scp* | 0.2169 | 0.1974 | 0.0195 | 0.046772 |
| *mcp* | 8.734 | 7.781 | 0.953 | 0.058273 |
| *icp* | 0.3039 | 0.2579 | 0.046 | 0.080079 |
| *xscp* | 0.1151 | 0.09904 | 0.01606 | 0.087811 |
| *mfb* | 0.03607 | 0.03648 | -0.00041 | 0.907511 |
| *Rbd* | 0.02352 | 0.01893 | 0.00459 | 0.092131 |
| *VLA* | 0.01148 | 0.009778 | 0.001702 | 0.718495 |
| *vsc* | 0.1472 | 0.1333 | 0.0139 | 0.156406 |
| *Med* | 0.2254 | 0.1949 | 0.0305 | 0.162726 |
| *MedDL* | 0.08267 | 0.07437 | 0.0083 | 0.246710 |
| *MedL* | 0.03522 | 0.02896 | 0.00626 | 0.162726 |
| *Lat* | 0.3429 | 0.3100 | 0.0329 | 0.297566 |
| *LatPC* | 0.06174 | 0.05378 | 0.00796 | 0.396825 |
| *SMV* | 0.04937 | 0.04433 | 0.00504 | 0.495670 |
| *DC* | 0.5019 | 0.4380 | 0.0639 | 0.125039 |
| *VCA* | 0.5901 | 0.5636 | 0.0265 | 0.718495 |
| *VCP* | 0.1957 | 0.1823 | 0.0134 | 0.718495 |
| *IntA* | 0.3124 | 0.2875 | 0.0249 | 0.297566 |
| *IntDL* | 0.08433 | 0.07611 | 0.00822 | 0.582147 |
| *IntP* | 0.1621 | 0.1396 | 0.0225 | 0.001793 |
| *IntPPC* | 0.07289 | 0.06189 | 0.011 | 0.025998 |
| *das* | 0.04226 | 0.03600 | 0.00626 | 0.400397 |

**SUPPLEMENTARY FIGURE LEGENDS**

**Supplementary Figure 1: In-situ hybridisation expression of *Tead1* mRNA in the developing embryonic brain**.

Sagittal sections taken from the E15.5 (A) and E18.5 (B) embryo, highlighting expression of *Tead1* mRNA in the developing cerebellum. Importantly, *Tead1* mRNA can be seen within the nascent rhombic lip of the E15.5 cerebellar anlage (A’), as well as within the EGL of the E18.5 cerebellum (B’). All images taken from the Allen Brain Atlas^1, 2^ – A, A’: <https://developingmouse.brain-map.org/experiment/show/100054550>; B, B’: <https://developingmouse.brain-map.org/experiment/show/100072097>

**Supplementary Figure 2: TEAD1 is expressed by unipolar brush cells**.

Mid-sagittal section taken from a P20 wild-type cerebellum, showing expression of TEAD1 (red) and TBR2 (green). Nuclei were labelled with DAPI (grey). Expression of TEAD1 was coincident with TBR2 in some cells (arrowheads), however, there were cells which were positive for only TEAD1 (double arrows) or for TBR2 (arrows) evident also. Scale bar (in D) represents 20 µm for all panels.

**Supplementary Figure 3: Volumetric analysis of adult *Tead1* cerebella.**

(A – C) Volumetric analyses of cerebella from adult control (grey; A) and cHet (orange; B) mice. Coronal sections shown represent an evenly spaced rostral to caudal progression of structural MRI B0 images, with the panels in C showing the cHet images superimposed over the control, revealing that the cHet sections are smaller. (D) Quantification of total cerebellar volume revealed that cHet cerebella were significantly smaller than controls. Likewise numerous cerebellar sub-regions were reduced in size. A full volumetric quantification of all 46 cerebellar sub-regions is shown in Supplementary Table 2. Displayed statistics represent a student’s *t*-test with Holm-Sidak correction. Scale bar represents 2 mm for all panels in A – C.

**Supplementary Figure 4: Increase in SOX2-expressing cell number in the IGL in the absence of *Tead1***.

Mid-sagittal sections of control (A-C) and cKO (D-F) cerebella at P20, revealing the expression of SOX2 (green); nuclei were labelled with DAPI (grey). In control cerebella, SOX2^+^ soma were distributed relatively uniformly throughout the cerebellum, with monolayer organisation of BG soma evident. In comparison, cKO cerebella exhibited significantly more SOX2^+^ soma throughout the cerebellum, with BG monolayer formation largely absent. While the number of SOX2-expressing cells within the Purkinje cell layer of the cKO was not significantly different from the control (G), there were significantly more Sox2^+^ soma in the mutant overall, especially within the IGL (H). Scale bar (in F) represents 20 µm for all panels. ns = not significant, * *p* < 0.05; one-way ANOVA.

**Supplementary Figure 5: Purkinje neuron monolayer formation is disturbed in the absence of *Tead1*.**

Mid-sagittal sections of control (A – C) and cKO (D – F) cerebella at P20, revealing the expression of parvalbumin (green); nuclei were labelled with DAPI (grey). In control cerebella, formation of the PCL monolayer (dotted lines demarcate the PCL) was evident. In comparison, cKO cerebella exhibited disturbances with PN monolayer formation. In contrast, no obvious differences in ML interneuron localisation within the ML (arrowheads in A – F) was observed. Scale bar (in F) represents 20 µm for all panels.

1. Science AIfB. Allen Mouse Brain Atlas [100054550]. 2004.

2. Science AIfB. Allen Mouse Brain Atlas [100072097]. 2004.
