# Supplementary figures and images for: The Hippo effector TEAD1 regulates postnatal murine cerebellar development
Source: Brain Struct Funct. 2025 Mar 10;230(3):42. doi: 10.1007/s00429-025-02903-x (PMC11893647; doi:10.1007/s00429-025-02903-x)

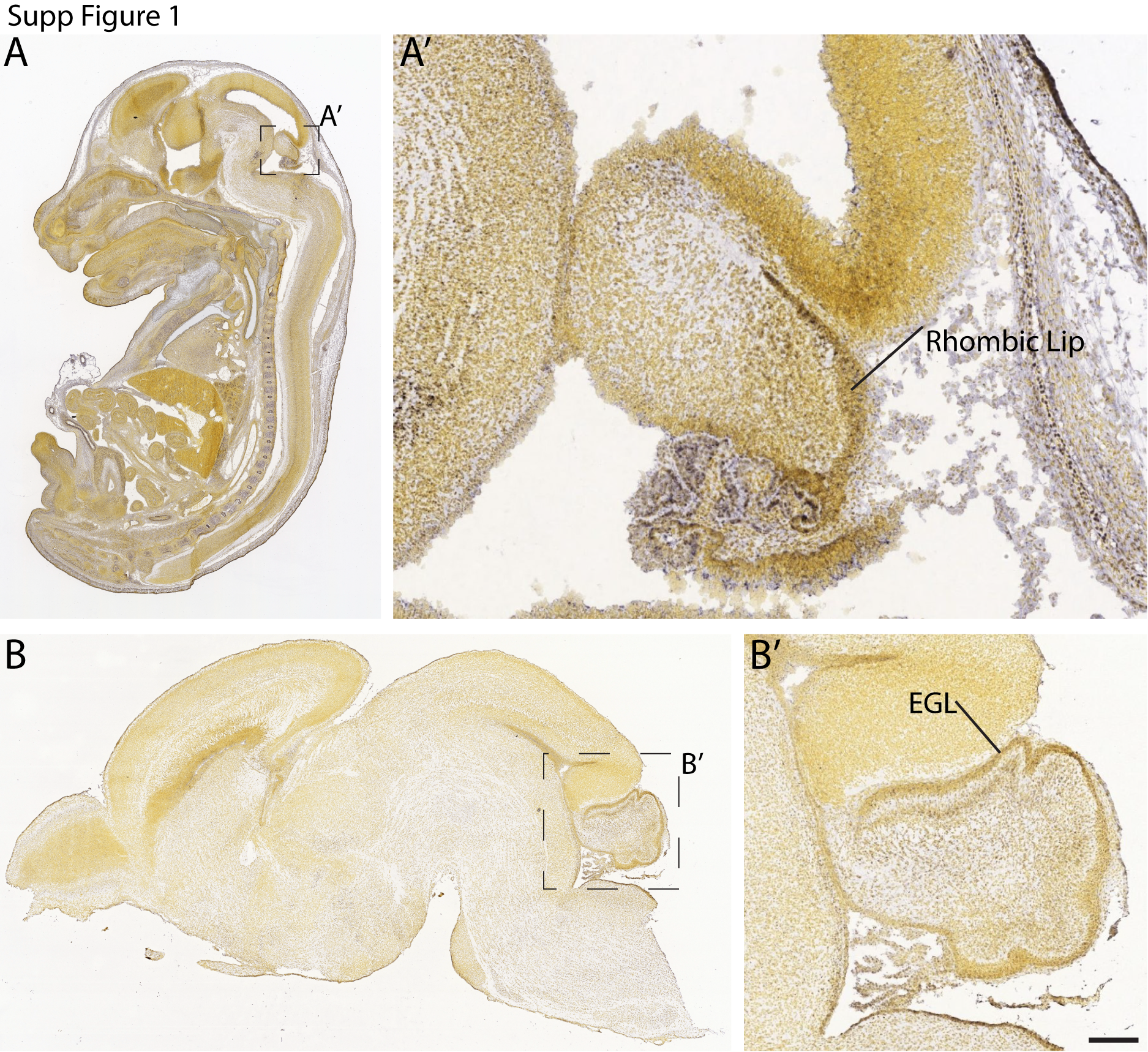

Supplement: Supplementary file 2 — Supplementary file2 (TIF 17776 kb) [file 429_2025_2903_MOESM2_ESM.tif]

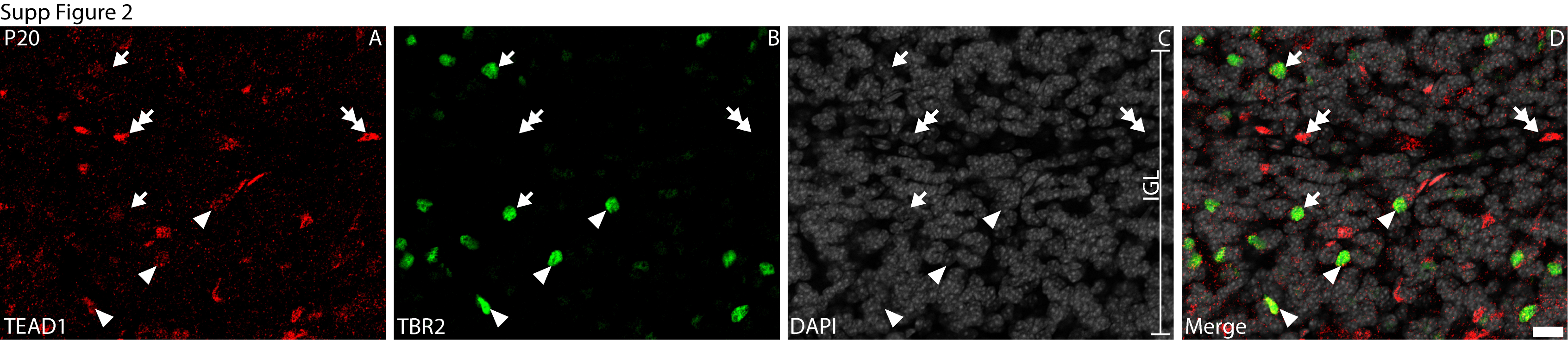

Supplement: Supplementary file 3 — Supplementary file3 (TIF 12118 kb) [file 429_2025_2903_MOESM3_ESM.tif]

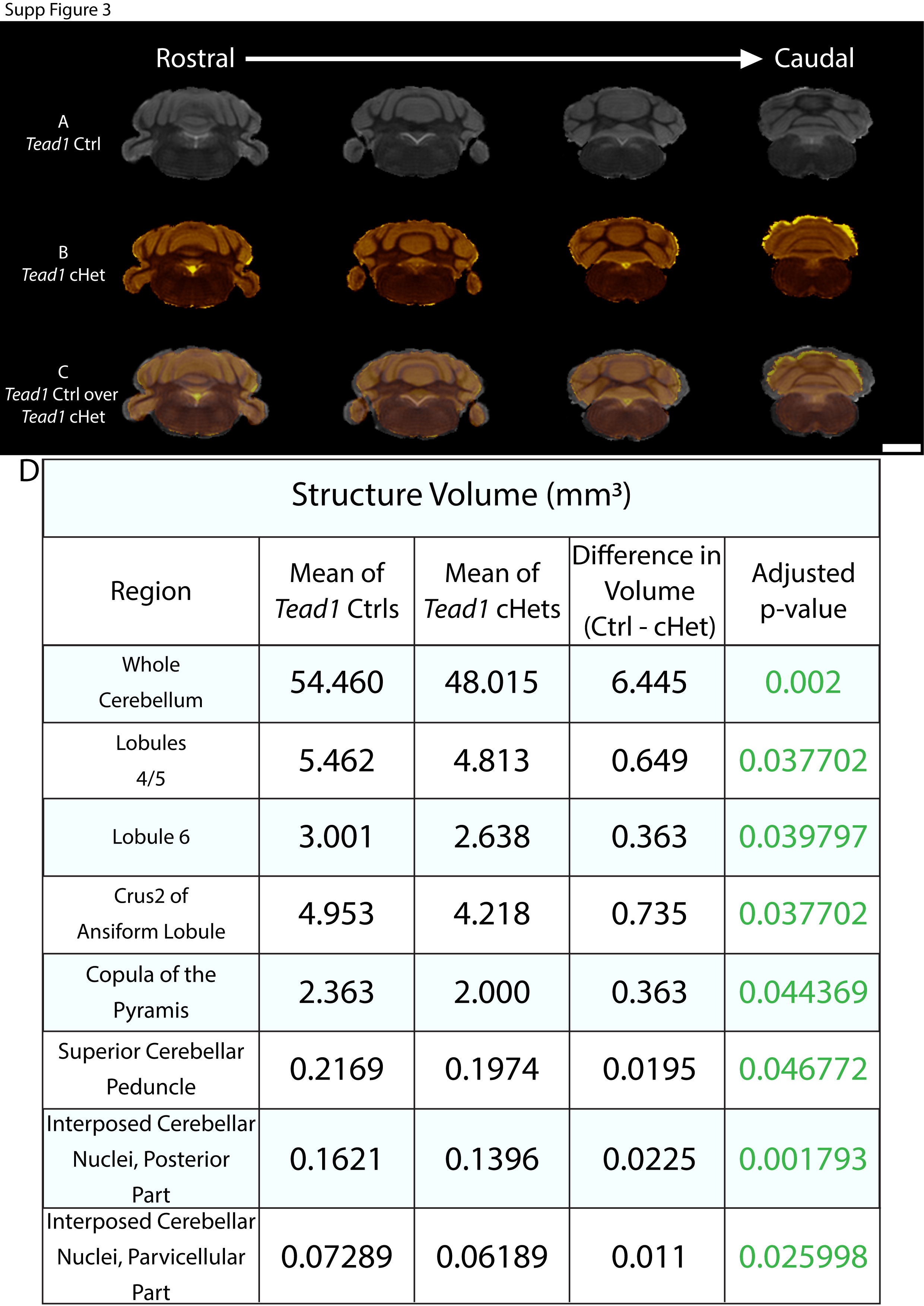

Supplement: Supplementary file 4 — Supplementary file4 (TIF 28976 kb) [file 429_2025_2903_MOESM4_ESM.tif]

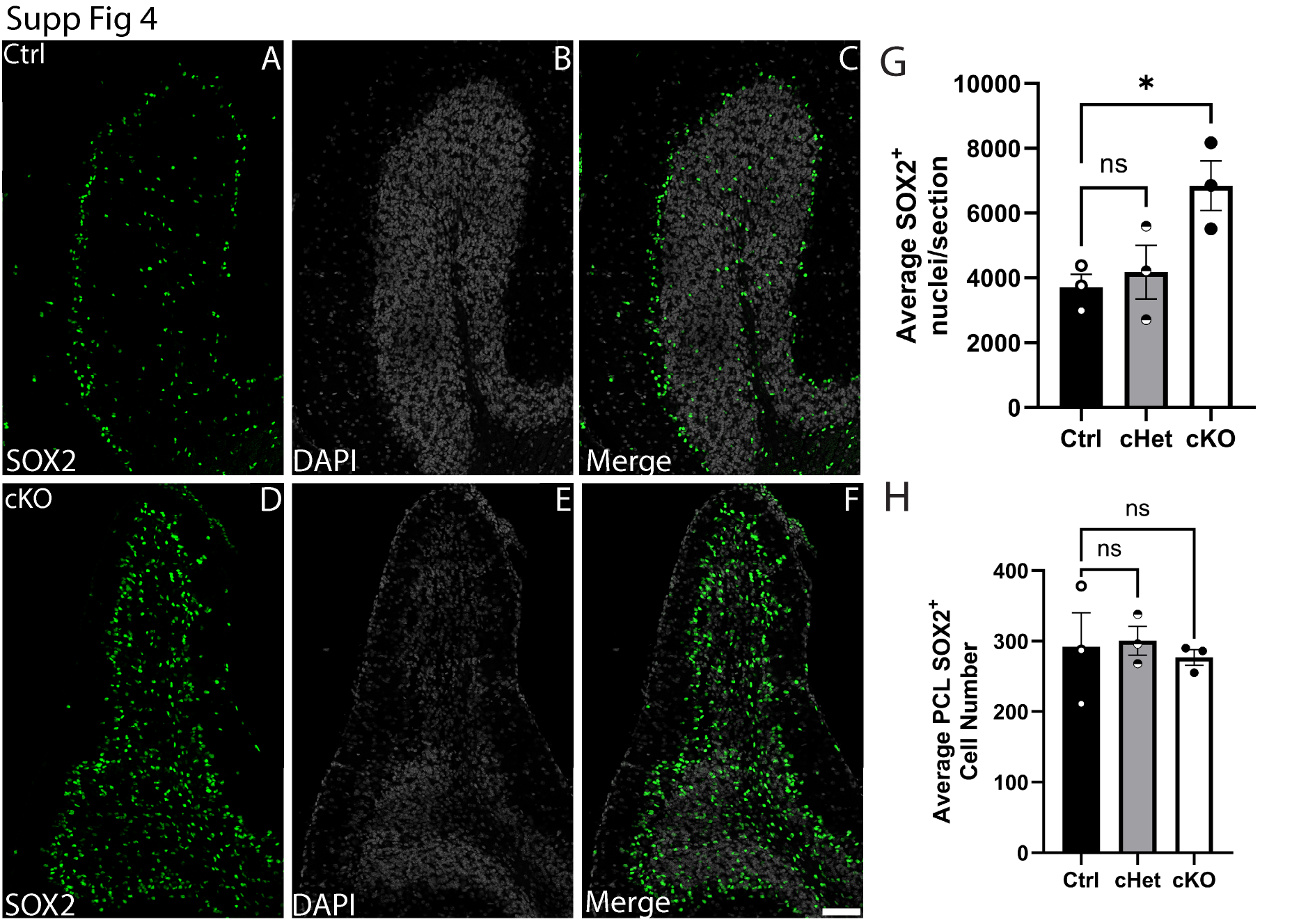

Supplement: Supplementary file 5 — Supplementary file5 (TIF 9927 kb) [file 429_2025_2903_MOESM5_ESM.tif]

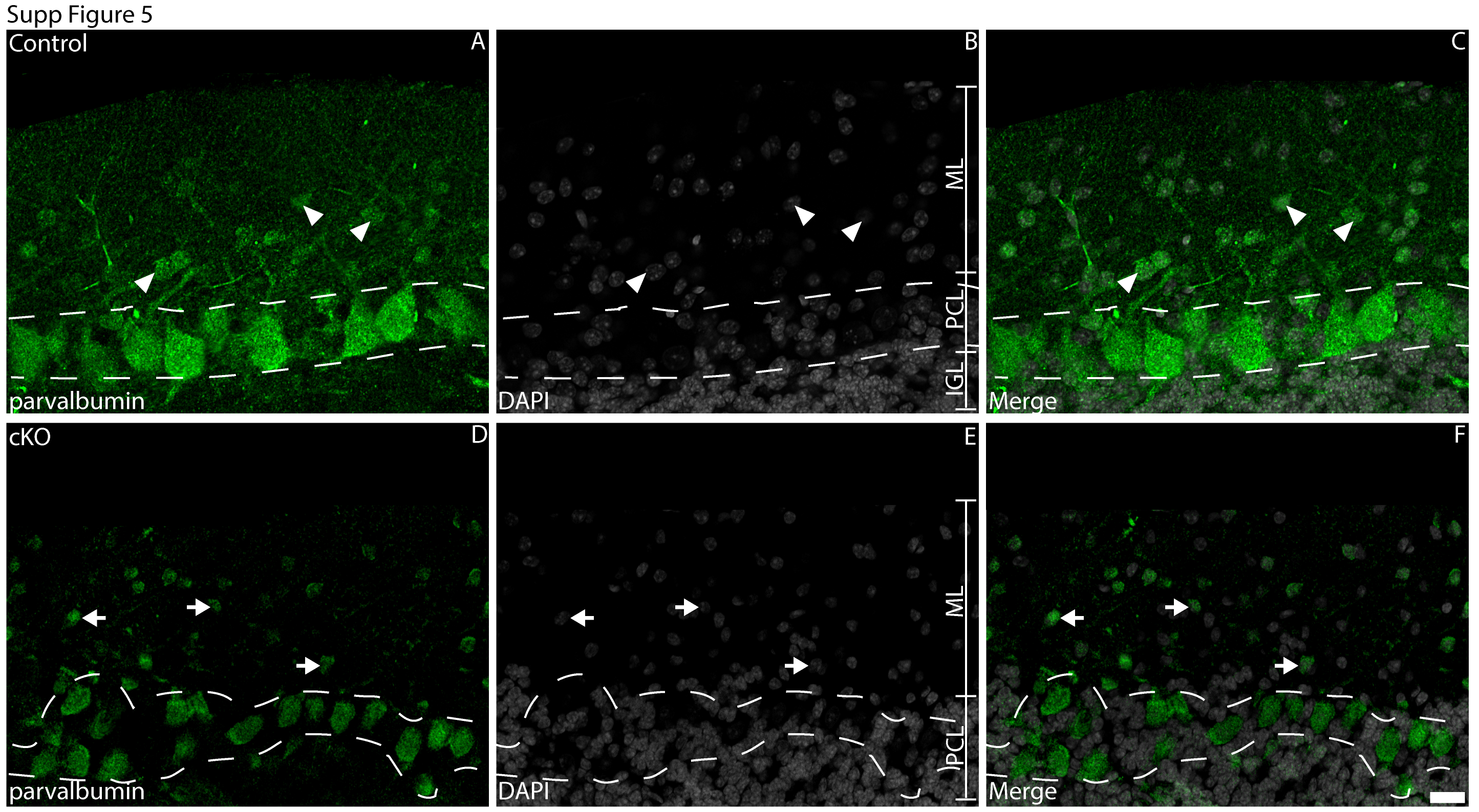

Supplement: Supplementary file 6 — Supplementary file6 (TIF 21127 kb) [file 429_2025_2903_MOESM6_ESM.tif]
